# Phosphorylation of OGFOD1 by Cell Cycle-Dependent Kinase 7/9 Enhances the Transcriptional Activity of RNA Polymerase II in Breast Cancer Cells

**DOI:** 10.3390/cancers13143418

**Published:** 2021-07-08

**Authors:** Han-Teo Lee, Il-Hwan Lee, Jae-Hwan Kim, Sangho Lee, Sojung Kwak, Min-Young Suh, In-Young Hwang, Bu-Gyeong Kang, Sun-Shin Cha, Byung-Il Lee, Sang-Eun Lee, Jinmi Choi, Jae-Seok Roe, Eun-Jung Cho, Hong-Duk Youn

**Affiliations:** 1 National Creative Research Center for Epigenome Reprogramming Network, Department of Biomedical Sciences, Ischemic/Hypoxic Disease Institute, Seoul National University College of Medicine, Seoul 03080, Korea; leehanteo@snu.ac.kr (H.-T.L.); ianlee.dba@snu.ac.kr (I.-H.L.); imslice7@snu.ac.kr (J.-H.K.); dltkdgh89@snu.ac.kr (S.L.); sojung.kwak@gmail.com (S.K.); viamee@snu.ac.kr (M.-Y.S.); inyoung.hwang@embl.de (I.-Y.H.); jcws0517@skku.edu (J.C.); 2 Department of Molecular Medicine & Biopharmaceutical Sciences, Graduate School of Convergence Science and Technology, Seoul National University, Seoul 03080, Korea; 3 Department of Chemistry & Nanoscience, Ewha Womans University, Seoul 03760, Korea; mybirth404@naver.com (B.-G.K.); chajung@ewha.ac.kr (S.-S.C.); 4 Research Institute, National Cancer Center, Goyang-si 10408, Korea; bilee@ncc.re.kr; 5 Cardiology Asan Medical Center, University of Ulsan College of Medicine, Seoul 05505, Korea; sangeunlee.md@gmail.com; 6 College of Pharmacy, Sungkyunkwan University, Suwon 16419, Korea; echo@skku.edu; 7 Department of Biochemistry, College of Life Science and Biotechnology, Yonsei University, Seoul 03722, Korea; jroe@yonsei.ac.kr

**Keywords:** OGFOD1, RNA polymerase II, transcriptional regulation, cell cycle-dependent kinase, tumorigenesis

## Abstract

**Simple Summary:**

Among the causes of accelerating cancer properties, dysregulated transcription is considerably prominent in many cancers. However, it is difficult to target transcriptional machineries due to their fundamental importance. Compared to breast cancer cell lines, we found that OGFOD1 aggravates cancers by enhancing RNA polymerase II transcriptional activity and it is improved by cell cycle-dependent kinases. Overall, we uncovered the novel mechanism for how OGFOD1 maliciously functions in breast cancers, suggesting it as a rational cancer treatment target protein.

**Abstract:**

2-oxoglutarate and iron-dependent oxygenase domain-containing protein 1 (OGFOD1) expression is upregulated in a variety of cancers and has been related to poor prognosis. However, despite this significance to cancer progression, the precise oncogenic mechanism of OGFOD1 is not understood. We demonstrated that OGFOD1 plays a role in enhancing the transcriptional activity of RNA polymerase II in breast cancer cells. OGFOD1 directly binds to the C-terminal domain of RNA polymerase II to alter phosphorylation status. The elimination of OGFOD1 resulted in decreased tumor development. Additionally, cell cycle-dependent kinase 7 and cell cycle-dependent kinase 9, critical enzymes for activating RNA polymerase II, phosphorylated serine 256 of OGFOD1, whereas a non-phosphorylated mutant OGFOD1 failed to enhance transcriptional activation and tumor growth. Consequently, OGFOD1 helps promote tumor growth by enhancing RNA polymerase II, whereas simultaneous phosphorylation of OGFOD1 by CDK enzymes is essential in stimulating RNA polymerase II-mediated transcription both in vitro and in vivo, and expression of target genes.

## 1. Introduction

2-oxoglutarate and iron-dependent oxygenase domain-containing protein 1 (OGFOD1) is a prolyl hydroxylase, with well-conserved homologs from yeast to humans. Under stress conditions, OGFOD1 is incorporated into stress granules and functions as a pro-apoptotic regulator by altering phosphorylation of eukaryotic translation initiation factor 2a (eIF2a) [1]. Additionally, loss of OGFOD1 increases resistance to cellular death in ischemia [2], and recent studies revealed that OGFOD1 catalyzes the hydroxylation of the small ribosomal protein S23 (RPS23), which enhances translational termination efficiency [3,4,5]; furthermore, OGFOD1 regulates alternative RNA splicing [6]. These results indicate that OGFOD1 dysfunction could skew cell growth. Elevated OGFOD1 levels have been reported in a variety of cancers such as chronic lymphocytic leukemia, breast cancer, and laryngeal papilloma and are associated with abnormal cell proliferation, dysregulated cell cycle, and poor prognosis [7,8,9]. Myc, a representative oncogene, can also induce OGFOD1 expression via the aryl hydrocarbon receptor in colon cancer [10]. However, despite a significant function being indicated in cancer progression, the precise mechanism of how OGFOD1 exacerbates cancer remains elusive.

RNA polymerase II is a central enzyme in the transcription of DNA to RNA. RNA polymerase II is a multiprotein complex that comprises 12 subunits. Rpb1 is the largest subunit with a carboxyl terminal domain (CTD) containing distinct heptapeptide repeats, Tyr-Ser-Pro-Thr-Ser-Pro-Ser (YSPTSPS). This domain is well conserved in many organisms and is phosphorylated following transcriptional initiation and elongation [11,12]. At first, hypophosphorylated polymerase forms a pre-initiation complex on the promoter region with general initiation factors and a mediator complex. Subsequently, cell cyclin-dependent kinase 7 (CDK7) phosphorylates serine 5 to initiate transcription [13]; phosphorylation of serine 2 is then facilitated by CDK9 to stimulate the transition from initiation to elongation [14] and enables recruitment of additional transcriptional modulators. These dynamic events may be sophisticatedly orchestrated by multiple machineries to regulate proper gene expression.

A key feature of cancer is dysregulated gene expression control. Abnormal transcription occurs in response to a subset of oncogenes, perturbed metabolites, and transcriptional machineries [15]. Several studies in cancer development induction have demonstrated that CDK-dependent transcriptional addiction is necessary for accelerating cancer growth in small-cell lung cancer, triple-negative breast cancer, ovarian cancer, and thyroid cancer [16,17,18,19]. Therefore, the inhibition of upregulated gene expression has been attempted as a cancer therapy. However, as transcriptional machinery is essential, targeting CDK activity is difficult to achieve in cancer therapy. Thus, it is necessary to understand the detailed mechanism that is enhanced and relevant in cancer.

OGFOD1 is increased in many cancers and regulates both transcription and translation; therefore, there may be a role for OGFOD1 in cancer development. Here, we identified a novel function for nuclear OGFOD1 in the enhancement of RNA polymerase II transcriptional activity and how this is governed by CDK7 activity in breast cancer cells.

## 2. Materials and Methods

### 2.1. Cell Culture

Human breast carcinoma cell line MDA-MB-231, HCC1954, T47D, MCF7 and human embryonic kidney cell line 293T, and non-tumorigenic epithelial cell line MCF10A were acquired from American Type Culture Collection (Manassas, VA, USA) and maintained at low passage (<20). MDA-MB-231, T47D, and HEK293T cell lines were cultured in Dulbecco’s modified Eagle medium (DMEM) with 10% (*v/v*) fetal bovine serum (FBS) and penicillin–streptomycin at 37 °C in a humidified atmosphere of 5% CO_2_. A total of 0.01 mg/mL human insulin was added for MCF7 cell line. For HCC1954 cell lines, Roswell Park Memorial Institute (RPMI) 1640 medium with 10% (*v/v*) FBS and penicillin–streptomycin was used. DMEM/F-12 with 5% (*v/v*) horse serum, 0.1 μg/mL cholera toxin, 0.5 μg/mL hydrocortisone, 20 ng/mL EGF, and 10 μg/mL insulin was used for MCF10A.

### 2.2. Plasmids

Full-length and truncated cDNA constructs of OGFOD1 were cloned into expression vectors pCAG, pGEX-4T-1, and pRSET-B. For lentivirus-mediated gene transduction, the cDNA constructs encoding OGFOD1 were subcloned into Gateway donor vector pDONR-221 and subsequently into the lentiviral destination vector pLX301 via Gateway Technology (Thermo Fisher Scientific, Waltham, MA, USA). Full-length Flag-tagged RNA Polymerase II (Flag-Pol II.FL) and deletion constructs (Flag-Pol II.ΔCTD) were gifts from Benjamin Blencowe (Addgene plasmid #35175 and #35176) [20]. pSpCas9n(BB)-2A-GFP (PX461) was a gift from Feng Zhang (Addgene plasmid #48140) [21]. DNA coding for human CDK7, cyclin H, MNAT1, CDK9, and cyclin T was PCR amplified from human cDNA and cloned into the pCAG mammalian expression vector. All protein-coding sequences were verified by sequencing.

### 2.3. CRISPR/Cas9-Mediated Gene Knockout

CRISPR/Cas9-mediated *OGFOD1* gene knockout was conducted as described previously [21]. Single guide RNA (sgRNA) sequences targeting the second exon of *OGFOD1* gene were designed using the CRISPR Design Tool (http://tools.genome-engineering.org, 20 April 2017). The sgRNA oligonucleotides (Table S1) containing BbsI sticky ends were synthesized, annealed, phosphorylated, and ligated into the BbsI-digested PX461 vector. Two sgRNA-containing plasmids were transfected into MDA-MB-231 cells using Lipofectamine 3000 reagent (Thermo Fisher Scientific, #L3000-015). A GFP-expressing single cell was isolated using BD cell sorter Aria™II (BD Biosciences, San Jose, CA, USA). Using specific primers for the second exon of *OGFOD1* gene, genomic DNA was isolated from each cell and PCR amplified (Table S1). Amplified PCR product was gel purified using DNA Gel Extraction Kit (Corning, AP-GX-250) and sequenced. For Western blotting, cells were lysed and then probed with the anti-OGFOD1 antibody.

### 2.4. Proximity-Dependent Labeling

pEJS578_DD-dSpyCas9-mCherry-APEX2 was a gift from Erik Sontheimer (Addgene plasmid #108570) [22]. APEX2 was PCR amplified and cloned into pCAG-OGFOD1 construct. Proximity labeling assay was performed as previously described [23]. OGFOD1-APEX2 was transiently transfected using polyethylenimine. After 24 h post transfection, the medium was changed with 500 μM biotin-phenol (Sigma-Aldrich, St. Louis, MO, USA, SML2135) and incubated at 37 °C for 30 min under 5% CO_2_. Subsequently, H_2_O_2_ was added to a final concentration of 1 mM and the plate gently agitated for 1 min. The reaction was quenched by washing three times with quenching buffer (5 mM Trolox, Sigma-Aldrich, #238813; 10 mM sodium ascorbate, Sigma-Aldrich, A7631 in PBS). Biotin-labeled cells were harvested and lysed in lysis buffer (25 mM Tris-Cl, pH 8.0, 150 mM NaCl, 0.1% (*v/v*) NP-40, 10% (*v/v*) glycerol, 1 mM ethylenediaminetetraacetic acid (EDTA) with protease inhibitors, phosphatase inhibitors, 5 mM Trolox, and 10 mM sodium ascorbate) and sonicated. The supernatant was collected after centrifugation at 14,000 rpm at 4 °C for 10 min. Streptavidin-conjugated agarose beads were washed twice with lysis buffer and incubated with cleared supernatant overnight at 4 °C. Beads were washed twice with lysis buffer, once with 1 M KCl, once with 0.1 M Na_2_CO_3_, once with 2 M urea in 10 mM Tris-Cl, pH 8.0, and twice with lysis buffer. Biotinylated proteins were eluted with elution buffer (2% sodium dodecyl sulfate (SDS), 30 mM biotin, 6 M urea, 2 M thiourea, 5× dye, 100 mM NaCl) via incubation at room temperature for 15 min and then at 96 °C for 15 min.

### 2.5. Silver Staining

Samples were separated by polyacrylamide 4–12% gel electrophoresis (PAGE) (Invitrogen, Middlesex County, MA, USA). The gel was fixed with fixing buffer (40% (*v/v*) ethanol, 10% (*v/v*) acetic acid in distilled water) for 1 h. The fixed gel was washed twice with 30% (*v/v*) ethanol for 20 min and then twice with distilled water for 20 min. Proteins were sensitized for 1 min in 0.02% (*w*/*v*) Na_2_S_2_O_3_ and washed twice with distilled water for 30 s. The gel was incubated with ice-chilled 0.1% (*w*/*v*) AgNO_3_ for 20 min and washed twice with distilled water for 30 s. Gels were developed with 3% (*w*/*v*) Na_2_O_3_ and 0.05% (*w*/*v*) formaldehyde until gel turned yellow. Staining was terminated by immersion in 5% acetic acid for 5 min, followed by washing three times with distilled water for 30 s. The stained gel was stored at 4 °C, protected from light.

### 2.6. Protein Purification

Glutathione S-transferase (GST)-tagged proteins were expressed in *E. coli* DH5α, and 6×His-tagged hOGFOD1 (full-length wild-type, S256A mutant, and several truncated mutants) were purified from *E. coli* BL21(DE3). The expression of GST or His-tagged proteins was induced by the addition of IPTG to a final concentration of 0.1–0.5 mM at 20 °C overnight or at 37 °C for 2–3 h when the optical density (OD 600 nm) of the culture reached 0.5–0.6. Cells were harvested via centrifugation and resuspended in lysis buffer (50 mM Tris-Cl, pH 7.5, 150 mM NaCl, 1.5 mM MgCl_2_, and 0.2 mM phenylmethylsulfonyl fluoride (PMSF)). Cells were lysed by sonication and the addition of TritonX-100 to 1% (*v/v*); insoluble materials were pelleted by centrifugation at 12,000 rpm for 15 min. The supernatant was mixed with pre-equilibrated glutathione sepharose 4B (GE Healthcare) or Ni-NTA agarose beads (QIAGEN, Hilden, Germany) at 4 °C for 1 h with gentle rotation. Beads were washed with lysis buffer three times and eluted with elution buffer (50 mM Tris-Cl, pH 8.0, 5% (*v/v*) glycerol, 10 mM reduced glutathione for GST-tagged proteins, or 250 mM imidazole for His-tagged proteins). Protein was concentrated using 10 kDa Amicon centricon devices, and protein concentration was analyzed using SDS-PAGE; the gel was stained with Coomassie Brilliant Blue (CBB).

### 2.7. Immunoprecipitation Assay

Transiently transfected 293T cells were lysed with lysis buffer (20 mM Tris-Cl, pH 7.5, 0.5% (*v/v*) NP-40, 150 mM NaCl, 1 mM PMSF, protease inhibitors, and phosphatase inhibitors). Cell lysates were incubated with indicated antibodies at 4 °C overnight and with protein G-agarose at 4 °C for 2 h. After three washes with lysis buffer, immunoprecipitated proteins were separated using SDS-PAGE, and Western blotting was conducted. For the endogenous immunoprecipitation (IP) assay, MDA-MB-231 cells were resuspended with lysis buffer and briefly sonicated. Total cell extract (1 mg) from MDA-MB-231 cells was incubated with indicated antibodies and IgG at 4 °C overnight. Protein complexes were further incubated with protein A/G PLUS-agarose for 2 h and washed three times with lysis buffer. Immunoprecipitates were separated with SDS-PAGE and probed with indicated antibodies.

### 2.8. In Vitro Binding Assay

GST pull-down assays were conducted in 500 μL of binding buffer (50 mM Tris-Cl, pH 7.5, 250 mM KCl, 0.05% (*v/v*) NP-40) containing bovine serum albumin (BSA) (2 μg/mL). GST and GST-OGFOD1 (0.5 μg) immobilized on glutathione sepharose beads were incubated with RNA polymerase II at 4 °C for 1 h. Precipitated protein complexes were washed three times with binding buffer without BSA, separated using SDS-PAGE, and analyzed via Western blotting.

### 2.9. In Vitro Phosphorylation Assay

In vitro radiolabeled phosphorylation assay was conducted in kinase reaction buffer composed of 50 mM HEPES-NaOH, pH 7.5, 3 mM MgCl_2_, 3 mM MnCl_2_, 3 μM sodium orthovandate, 1 mM dithiothreitol, 20 μM ATP (unlabeled), and 5 μCi γ-^32^P ATP. BL21(DE3)-expressed 6×His-hOGFOD1 was added as substrate and phosphorylated by CDK9/CyclinT1 (Thermo Fisher Scientific, Middlesex County, MA, USA, PV4131) and CDK7/CyclinH/MNAT1 (Thermo Fisher Scientific, PV3868) recombinant protein complexes. The assay was incubated at 30 °C for 1 h and stopped by the addition of 5× sample buffer. Samples were boiled for 10 min and were run in duplicate on separate PAGE gels, one for gel staining (CBB) and the other for autoradiogram analysis. Increasing concentrations of flavopiridol (Selleckchem, Houston, TX, USA, S1230) were used to inhibit CDK9/CyclinT1 and CDK7/CyclinH/MNAT1 kinase reactions.

### 2.10. mRNA Sequencing and Analysis

Using TRIzol RNA isolation reagent (Life Technologies, Carlsbad, CA, USA), total mRNA was isolated from parental and OGFOD1-knockout (OGFOD1^Δ/Δ^) MDA-MB-231 cells. RNA integrity was confirmed using an Agilent RNA 6000 Pico kit (Agilent, Santa Clara, CA, USA). Isolated RNA was used to prepare an mRNA sequencing library using TruSeq Stranded mRNA sample preparation kit (Illumina, San Diego, CA, USA). Briefly, mRNAs were isolated from 400 ng total RNA via RNA purification bead using polyA capture, followed by enzyme shearing. After the first- and second-strand cDNA synthesis, A-tailing and end repair were conducted for the ligation of proprietary primers that incorporated unique sequencing adaptors with an index for tracking Illumina reads from multiplexed samples run on a single sequencing lane. For each library, an insert size of approximately 200 bp was confirmed by a bioanalyzer using an Agilent DNA kit, and the quantification of the library was measured by real-time PCR using CFX96 real-time system (BioRad, Hercules, CA, USA). All the samples were sequenced on an Illumina NextSeq 500 Sequencer with a 75 bp paired-end High Output kit. The raw image data was transformed by base calling into sequence data and stored in FASTQ format. Reads of each sequencing sample were aligned to the human genome (GRCh37/hg19 genome assembly) using STAR (v2.4.0.1) [24] with the default settings. HOMER was used to quantify FPKM values and normalize genes defined from RefSeq transcripts. A heat map was generated using a Cluster3.0 and Java Treeview by the log_2_-centered values (ABS(Log_2_[Foldchange]) > 0.58, *p*-value < 0.05). The gene expression in parental and OGFOD1-knockout MDA-MB-231 cells was visualized using the Integrative Genomics Viewer (http://software.broadinstitute.org/software/igv, 13 March 2020). Enrichment analysis of large gene lists selected by log_2_ fold change was conducted using the DAVID web tool (https://david.ncifcrf.gov, 29 June 2020). The raw and processed RNA sequencing data from this work was submitted to the NCBI Gene Expression Omnibus (GEO) under accession number GSE160363.

### 2.11. Chromatin IP Assay

MDA-MB-231 cells were crosslinked by formaldehyde for 10 min at a final concentration of 1% in growth medium, followed by 5 min quenching with 125 mM glycine. Cells were washed twice with PBS and lysed with buffer A (5 mM PIPES, pH 8.0, 85 mM KCl, 0.5% (*v/v*) NP-40). The cytoplasmic fraction was separated by centrifugation at 5000 rpm for 5 min. Nuclei were resuspended in buffer B (50 mM Tris-Cl, pH 8.0, 10 mM EDTA, 1% (*v/v*) SDS) and incubated on ice for 10 min. Chromatin shearing was conducted with a Covaris S220 Focused-Ultrasonicator (Covaris, Woburn, MA, USA) using the following parameters: fill level 12, duty cycle 10, peak intensity power of 175, and cycles/burst 200, for 20 min. Sonicated lysates were clarified via centrifugation at 14,000 rpm at 4 °C for 10 min and the supernatant was retained; the DNA concentration was measured, and 50 μg was used for one IP. The supernatant was diluted 10-fold in IP buffer (16.7 mM Tris-Cl, pH 8.0, 167 mM NaCl, 0.01% (*v/v*) SDS, 1.1% (*v/v*) Triton X-100, 1.2 mM EDTA). IP samples were incubated with indicated antibodies at 4 °C overnight. Protein A/G-agarose was then added to each tube and incubated for another 2 h. The resulting immune complexes were washed once with low-salt washing buffer (20 mM Tris-Cl, pH 8.0, 150 mM NaCl, 0.1% (*v/v*) SDS, 1% (*v/v*) Triton X-100, 2 mM EDTA), once with high-salt washing buffer (20 mM Tris-Cl, pH 8.0, 500 mM NaCl, 0.1% (*v/v*) SDS, 1% (*v/v*) Triton X-100, 2 mM EDTA), once with LiCl buffer (10 mM Tris-Cl, pH 8.0, 0.25 M LiCl, 1% (*v/v*) NP-40, 1% (*w*/*v*) sodium deoxycholate), and twice with TE buffer (10 mM Tris-Cl, pH 8.0, 10 mM EDTA). Immune complexes were then eluted twice with 250 μL of elution buffer (1% (*v/v*) SDS, 0.1 M NaHCO_3_) with gentle rotation at room temperature. Then, 20 μL of 5 M NaCl was added to each sample, and de-crosslinking was conducted during an overnight incubation at 65 °C. The next day, free DNA was ethanol precipitated and analyzed.

### 2.12. Cell Proliferation, Invasion, and Migration Assay

For cell proliferation assay, 80% confluent cells were trypsinized, gently resuspended in a cell culture medium, and seeded as indicated cell counts per well in a six-well plate; cells were counted for 5 or 6 days at 3-day intervals. For wound-healing migration assay, 1 × 10^5^ cells per well were plated in a six-well plate. After 24 h incubation, the cell layer was scratched, and after 48 h post scratch, the average wound size was analyzed using Image J. For cell invasion assay, Matrigel (Corning, Corning, NY, USA #354234) was diluted in 1% (*v/v*) FBS-containing medium to a final concentration of 2 mg/mL. Diluted Matrigel, 50 μL, was added to the upper compartment of the cell culture insert (Falcon, #353097) and the 24-well plate, with insert, was immediately incubated at 37 °C for 30 min to solidify the liquid Matrigel. Cells (1 × 10^5^) in serum-free medium were gently added onto the Matrigel-coated membrane in the insert and then 500 μL of the cell culture medium with 10% (*v*/*v*) FBS was added. After 24 h incubation, cells were fixed with 4% (*w*/*v*) formaldehyde for 20 min, followed by staining with 0.1% (*w*/*v*) crystal violet in 10% (*v/v*) ethanol for an additional 20 min. Cell numbers were then counted under a microscope. For transwell cell migration assay, cells (0.5 × 10^5^) in serum-free medium were gently added onto the insert and then 500 μL of the cell culture medium with 10% (*v/v*) FBS was added to the wells. After 16 h incubation, cells that had migrated to the other side of the membrane were fixed and stained as above.

### 2.13. Lentivirus-Based shRNA Production and Expression

One day before transfection, 4 × 10^6^ 293FT cells were seeded into a 10 cm dish. Cells were transfected with either OGFOD1 shRNAs (Sigma-Aldrich, #SHCLNG-NM_018233) or control shRNA (shLuciferase) (Sigma-Aldrich, #SHC007) in serum-free DMEM containing viral packaging (psPAX2) and envelope (pMD2G) constructs using Lipofectamine 2000 reagent (Thermo Fisher, #11668019). After 18 h post-transfection, the cell culture medium was replaced with 10 mL of fresh medium, and cells were incubated for an additional 48 h. Lentivirus-containing supernatants were harvested via centrifugation at 2000 rpm for 10 min and residual cell debris removed using a 0.45 μm filter (Sartorius, #16555-K). To knock down the expression of OGFOD1 in MDA-MB-231 cells, 1 mL of lentivirus was infected with polybrene to 5 × 10^5^ cells and OGFOD1 mRNA and protein levels were monitored by RT-qPCR and Western blot analysis, respectively. The full western blots can be found at Figures S6–S15. 

### 2.14. Animal Studies

Six-week-old female NOD/SCID mice were used for tumor progression in a subcutaneous xenograft experiment using 5 × 10^6^ cells (MDA-MB-231, parental; MDA-MB-231/OGFOD1^Δ/Δ^;MDA-MB-231/OGFOD1^Δ/Δ^/WT;MDA-MB-231/OGFOD1^Δ/Δ^/S256A) (*n* = 4 per group). Each week, tumor length (L) and width (W) were measured with calipers, and the volume calculation was obtained by the formula V = (W × W × L)/2.

### 2.15. Statistical Analysis

Comparison of two groups for statistical significance analysis was carried out using the Student’s t-test. To analyze multiple groups, one-way ANOVA with Tukey’s multiple comparison test was conducted in Prism 7 software (GraphPad Software Inc., La Jolla, CA, USA). Data are presented as mean ± standard deviation (SD) or ± standard error of the mean (SEM). The number of experimental replicates of experimental conditions is given in figure legends. The significant differences are indicated as the *p*-value: * *p* < 0.05, ** *p* < 0.01, *** *p* < 0.001.

## 3. Results

### 3.1. OGFOD1 Has an Important Role for Rapid Proliferation of Breast Cancer Cell Lines

**Figure 1:**
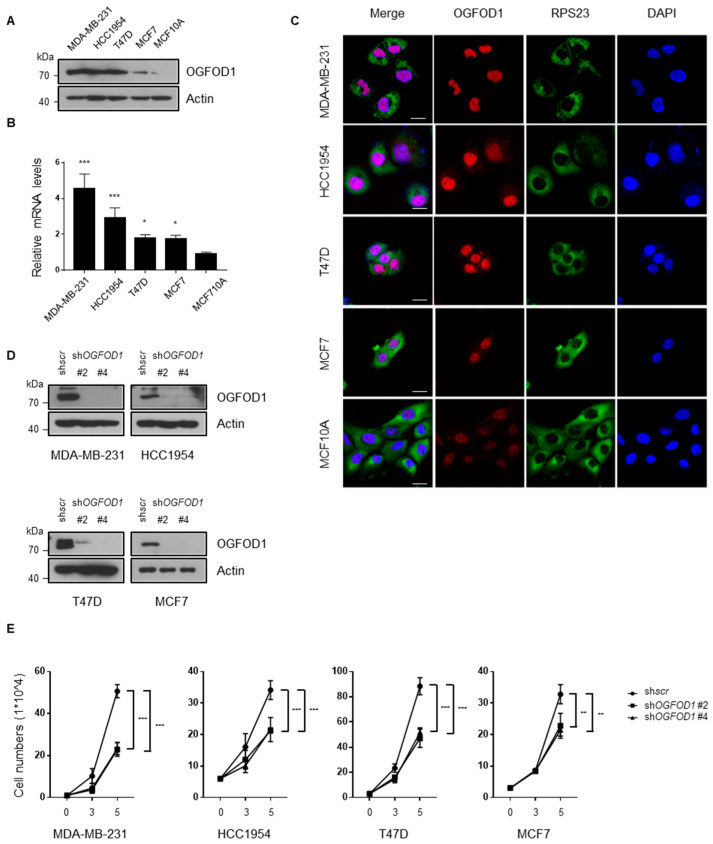
High level of OGFOD1 is important for proliferation. (**A**,**B**) Protein and mRNA levels of OGFOD1 in breast cancer cell lines and non-tumorigenic epithelial cell line. Protein levels were confirmed using Western blot assay. mRNA levels were compared using qRT-PCR. (**C**) Confocal images of OGFOD1 and RPS23 in breast cancer cell lines. OGFOD1 was stained with Alexa 568 (red), RPS23 was stained with Alexa 488 (green) and nuclei were stained with DAPI (blue); scale bar indicates 20 μm. (**D**) OGFOD1 knockdown in breast cancer cell lines. OGFOD1 was knocked down using lentiviral sh*RNAs* and was validated. shs*cr* was treated as a negative control. (**E**) Proliferation rate of breast cancer cell lines. Statistical data are presented as mean ± SD. Two-tailed unpaired Student’s *t*-test was conducted for statistical analysis (*n* = 4) (* *p* < 0.05, ** *p* < 0.01, *** *p* < 0.001).

In order to examine the role of OGFOD1 in breast cancer cell lines, we verified the expression level of OGFOD1 in MDA-MB-231, HCC1954, T47D, MCF7, and non-tumorigenic epithelial cell line MCF10A. Both protein and mRNA levels of OGFOD1 were highly expressed in breast cancer cells compared to epithelial cells (Figure 1A–C). Next, we sought to figure out whether upregulated OGFOD1 affects breast cancer property. Lentiviral sh*RNA* effectively knocked down OGFOD1 (Figure 1D). When OGFOD1 levels were reduced, all of the breast cancer cell lines exhibited a remarkably impeded proliferation rate (Figure 1E). These data indicated that a high level of OGFOD1 has the potential to promote rapid proliferation in breast cancer cell lines.

### 3.2. OGFOD1 Binds to the C-Terminal Domain of RNA Polymerase II and Attenuates Phosphorylation States

**Figure 2:**
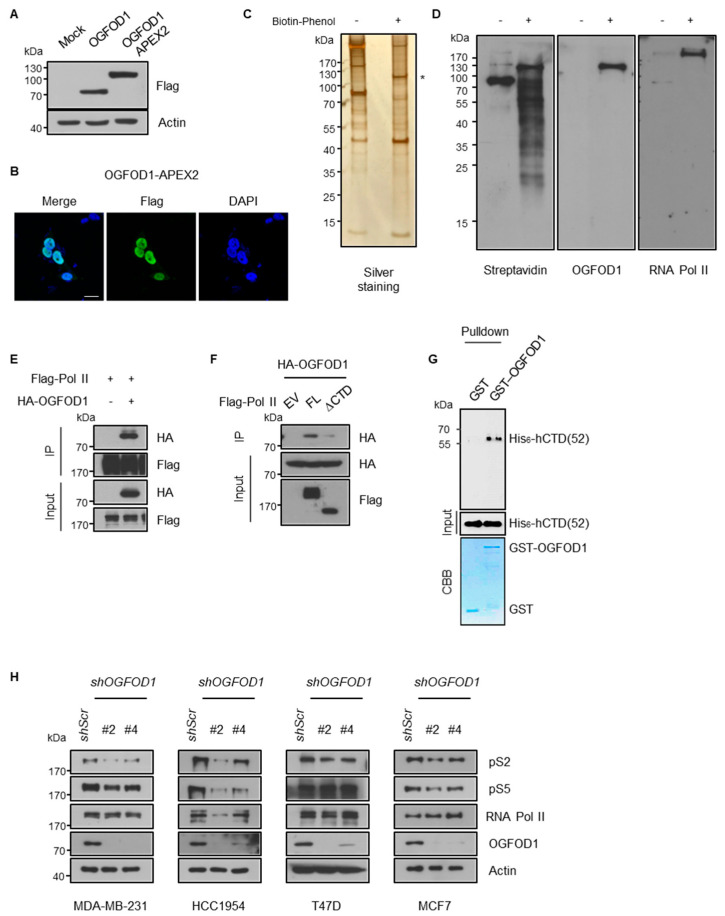
OGFOD1 binds to the C-terminal domain of RNA polymerase II and attenuates phosphorylation states. (**A**) Western blot images of OGFOD1-APEX2. The expression and molecular size were confirmed by SDS-PAGE. (**B**) Confocal microscopic images of OGFOD1-APEX2. Localization of OGFOD1-APEX2 was confirmed using immunofluorescence. Flag-tagged OGFOD1-APEX2 was stained with an anti-Flag antibody using Alexa 488 (green), and nuclei were stained with DAPI (blue); scale bar indicates 20 μm. (**C**) Silver staining of biotinylated proximal proteins. Proximal labeling was conducted with H_2_O_2_ and biotin-phenol. Biotin-labeled proteins were pulled down and visualized. Asterisk marks OGFOD1-APEX2. (**D**) Biotinylated proteins were detected using indicated antibodies. Streptavidin blot shows whole proteins biotinylated by OGFOD1-APEX2. (**E**,**F**) Immunoprecipitation of OGFOD1 and RNA polymerase II with or without C-terminal domain (CTD). Immuno-precipitation was conducted using anti-Flag antibody in HEK293T. (**G**) In vitro binding assay. Pulldown was conducted using glutathione agarose bead. The amounts of GST and GST-OGFOD1 proteins were confirmed using CBB. His_6_-hCTD was immunoblotted using CTD antibodies. GST lane used as a negative control. (**H**) Phosphorylation levels of RNA polymerase II in breast cancer cell lines with OGFOD1 KD.

We adopted a proximity-dependent labeling approach using an engineered ascorbate peroxidase (APEX2) system to determine how OGFOD1 affects cancer proliferation. APEX2 selectively allows biotinylation of proximal proteins at less than 20 nm distances, enabling an APEX2-tagged protein to mark any notable neighboring proteins [23]. We constructed APEX2-tagged OGFOD1 (OGFOD1-APEX2) (Figure 2A) and confirmed nuclear localization by immunostaining in HEK293T cell line to find target proteins that regulate cancer growth through OGFOD1 (Figure 2B). Using biotin-phenol and hydrogen peroxide treatment, OGFOD1-APEX2 biotinylated proximal proteins were pulled down with streptavidin beads. Purified proteins were detected by silver staining (Figure 2C). Interestingly, we found that RNA polymerase II was biotinylated using OGFOD1-APEX2 (Figure 2D), although RNA polymerase II was not detected via LC-MS/MS analysis. This evidence supports the possibility that OGFOD1 may cooperate with RNA polymerase II to control cellular proliferation. To verify this hypothesis, we immunoprecipitated OGFOD1 with RNA polymerase II to confirm their interaction and OGFOD1 clearly bound to RNA polymerase II (Figure 2E). Next, to identify the exact domain required for this interaction, we used full-length RNA polymerase II and C-terminal domain-deleted RNA polymerase II (∆CTD), as RNA polymerase II contains tandem heptapeptide repeats (YSPTSPS) in the C-terminal region and the phosphorylation status modulates transcriptional activity. OGFOD1 bound to full-length RNA polymerase II but not to ∆CTD (Figure 2F). Therefore, we tested if the CTD is critical for direct interaction using in vitro binding assays with purified recombinant GST-OGFOD1 and His_6_-hCTD (52) of RNA polymerase II. In vitro binding assay showed that OGFOD1 directly bound to the CTD of RNA polymerase II (Figure 2G).

### 3.3. OGFOD1 Alters Phosphorylation States of RNA Polymerase II

The phosphorylation of CTD mainly occurs on residues serine 2 and/or serine 5 of the repeated sequence by CDK7 and CDK9 and is a key component of transcriptional activity [25]. As OGFOD1 associates with CTD of RNA polymerase II, we hypothesized that OGFOD1 may affect RNA polymerase II transcriptional activity by altering these modifications. We knocked down OGFOD1 expression using sh*RNA* to understand the effect of OGFOD1 on transcriptional activity. Intriguingly, this resulted in a decrease in phosphorylation levels of both serine 2 and serine 5 in breast cancer cell lines (Figure 2H). Even though OGFOD1 was upregulated among breast cancer cell lines, the levels varied in each cell lines. The highest levels of OGFOD1 were detected in MDA-MB-231 and HCC1954, which showed a significant reduction in phosphorylation of CTD. T47D and MCF7, which have relatively low OGFOD1 levels, exhibited a lesser decrease in phosphorylation. Taken together, OGFOD1 collaborates with RNA polymerase II in the transcription process by affecting CTD phosphorylation.

### 3.4. Nuclear Localization of OGFOD1 Is Critical for Regulating RNA Polymerase II Activity

**Figure 3:**
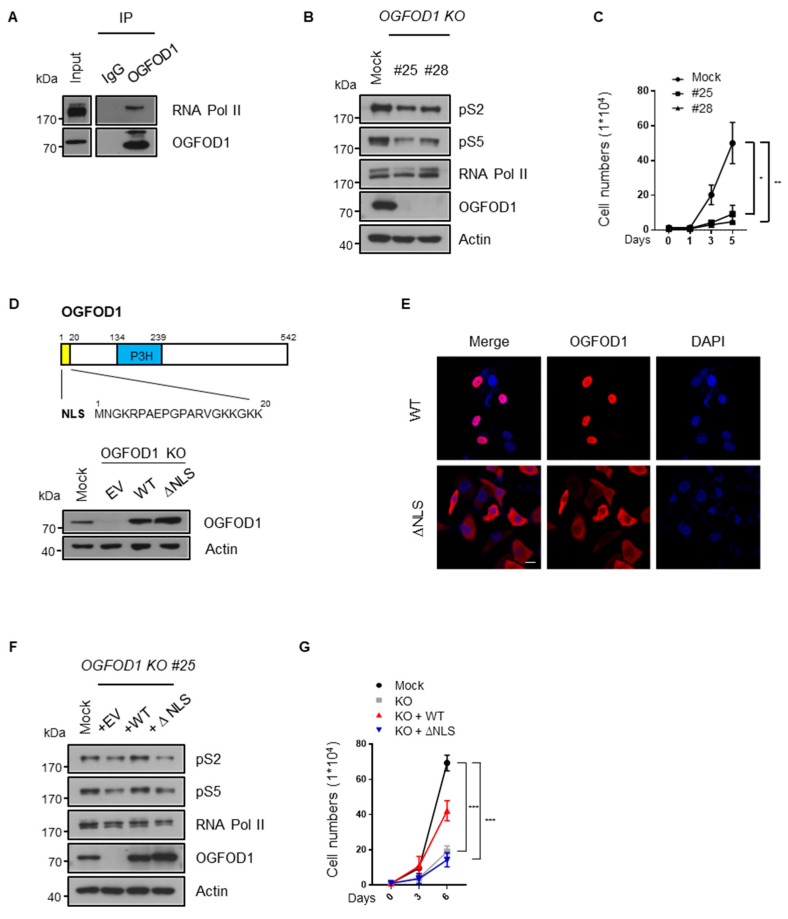
Nuclear OGFOD1 modulates RNA polymerase II activity. (**A**) Endogenous IP of OGFOD1 and RNA polymerase II in MDA-MB-231. Immunoprecipitation was performed using anti-OGFOD1 antibody. (**B**) Phosphorylation levels of CTD in OGFOD1 KO MDA-MB-231. (**C**) Proliferation rate with OGFOD1 KO. (**D**) A schematic structure of OGFOD1. A total of 1–20 amino acids indicated nuclear localization sequence (NLS). P3H: prolyl 3-hydroxylase domain. Wild-type or ∆NLS OGFOD1 restored at OGFOD1 knocked out MDA-MB-231. Western blot assay was performed to detect OGFOD1 protein levels. (**E**) Different localization of wild-type and ∆NLS OGFOD1 were verified using immunofluorescence images. OGFOD1s were stained with Alexa 568 (red), nuclei were stained with DAPI (blue). Scale bar indicates 20 μm. (**F**) Phosphorylation levels of backup cell lines. Wild-type or ∆NLS OGFOD1 restored in the OGFOD1 KO MDA-MB-231. Phosphorylation states of RNA polymerase II confirmed by indicated antibodies. (**G**) Proliferation rate of Mock, OGFOD1 KO, OGFOD1 KO + WT, OGFOD1 KO + ∆NLS. Statistical data are presented as mean ± SD. Two-tailed unpaired Student’s *t*-test was performed for statistical analysis. (*n* = 4), * *p* < 0.05, ** *p* < 0.01, *** *p* < 0.001.

Since we newly discovered the connection between OGFOD1 and RNA polymerase II and it is considered as a critical process of regulating cancer properties, we attempted to confirm their relation. To this end, OGFOD1-knockout cell lines (OGFOD1 KO) were established using the CRISPR/Cas9 system in MDA-MB-231 cells (Supplementary Figure S1) since MDA-MB-231 exhibits the highest level of OGFOD1, and we also previously reported that OGFOD1 is a critical regulator of proliferation and poor prognosis in this context [8]. First of all, we confirmed the interaction between OGFOD1 and RNA polymerase II in MDA-MB-231 (Figure 3A). As we expected, RNA polymerase II phosphorylation levels shrank according to OGFOD1 KO, resulting in decreased proliferation (Figure 3B,C). OGFOD1 is known to predominantly locate in nucleus due to a nucleus localization sequence (NLS). We generated an OGFOD1 mutant construct (∆NLS OGFOD1) with the nuclear localization sequence deleted to evaluate whether the nuclear position of OGFOD1 promotes an oncogenic potential. Wild-type or ∆NLS OGFOD1 were then overexpressed in the OGFOD1 KO cells (Figure 3D). Confocal analysis showed that wild-type OGFOD1 was localized in the nucleus but that ∆NLS OGFOD1 was excluded from the nucleus and remained in the cytosol (Figure 3E). Only wild-type OGFOD1 rescued phosphorylation levels of RNA polymerase II and ∆NLS OGFOD1 failed to rescue this ability in the knockout cell line (Figure 3F). Proliferation rate also recovered in proportion to the phosphorylation levels (Figure 3G). Taken together, these results indicate that the nuclear localization of OGFOD1 contributes to cancer development through RNA polymerase II.

### 3.5. OGFOD1 KO Reduces Metastatic Gene Expressions in MDA-MB-231

**Figure 4:**
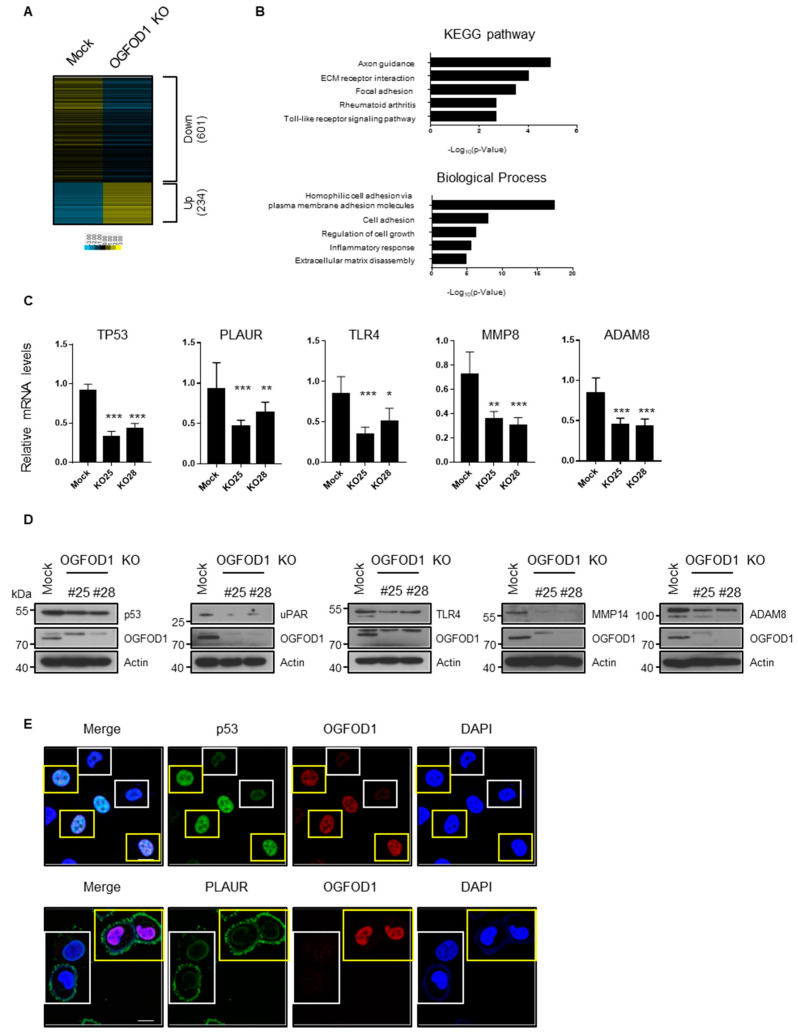
Altered genes by OGFOD1 knockout in MDA-MB-231. (**A**) Heatmap of altered genes by OGFOD1 knockout. Read per kilobase of transcript, per million mapped reads was transformed to log_2_ values by Spearman rank correlation. (**B**) The functional gene ontology (GO) analysis of downregulated genes. (**C**) qRT-PCR results of reduced genes. mRNA levels were confirmed in two other knockout clones. (**D**) Protein levels of reduced genes were confirmed in two other knockout clones. (**E**) Confocal images of p53 and PLAUR in mixed culture of wild-type (Mock) and OGFOD1 KO MDA-MB-231. Yellow and white boxes indicate wild type and OGFOD1 KO, respectively. Scale bar indicates 20 μm. * *p* < 0.05, ** *p* < 0.01, *** *p* < 0.001.

We conducted RNA sequencing from MDA-MB-231 cells to determine genes regulated by OGFOD1. Using OGFOD1 KO, we identified 234 upregulated genes and 601 downregulated genes (Figure 4A, Table S2). Gene ontology analysis indicated that downregulated genes were associated with metastasis, immune response, and receptor signaling (Figure 4B). Subsequent experiments confirmed that the levels of RNA and protein of targeted genes including *TP53*, *PLAUR*, *TLR4*, *MMP14*, and *ADAM8* were clearly decreased in the knockout cell line (Figure 4C–E, Figure S2). These data imply that OGFOD1 enhances not only proliferation but also oncogenesis in breast cancer cell lines.

### 3.6. CDK7 and CDK9 Phosphorylate Serine 256 of OGFOD1

**Figure 5:**
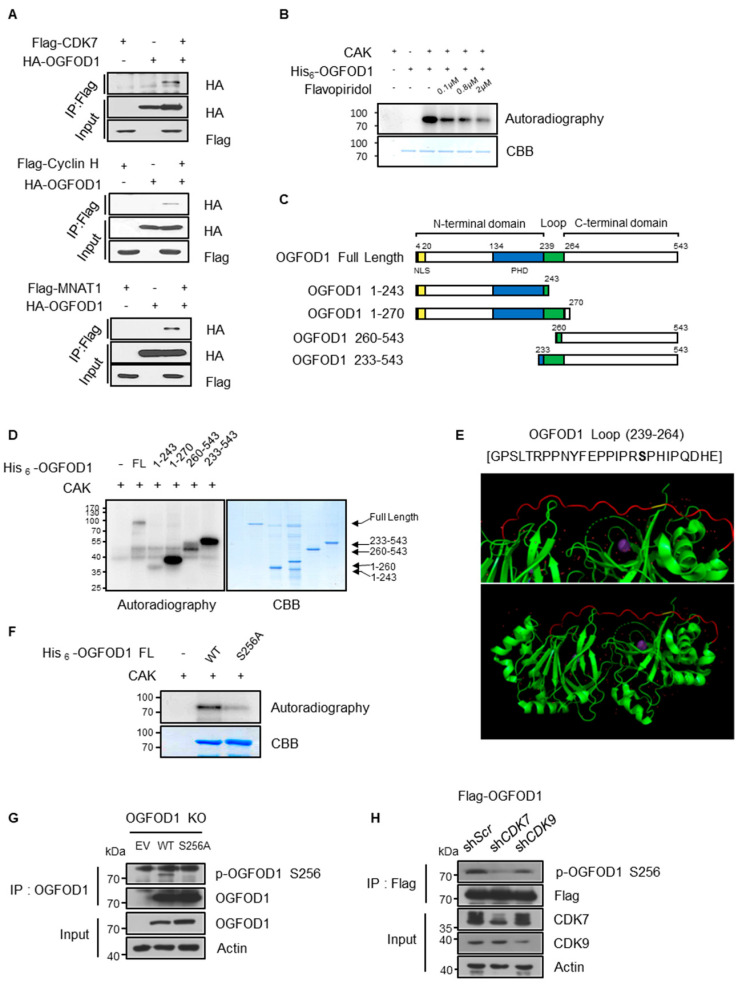
CDK7 and CDK9 phosphorylate serine 256 of OGFOD1 in vitro and in vivo. (**A**) Each of the CAK components (CDK7, cyclin H, and MNAT1) was co-expressed with HA-OGFOD1 in HEK293T. Immunoprecipitation of Flag-tagged proteins and co-precipitates was visualized by HA immunoblots. (**B**) *E. coli*-isolated recombinant His_6_-OGFOD1 was assessed via radioactive in vitro CDK7 kinase assay. Increasing concentrations of flavopiridol were used to inhibit kinase reaction. Radiolabeled His_6_-OGFOD1 was blotted and input protein was visualized by CBB staining. (**C**) Scheme of OGFOD1 full-length and truncated domains. Both N-, C-terminal domains of OGFOD1 with or without loop region were designed. (**D**) Radioactive kinase assay using His_6_-tagged truncated domains of OGFOD1. All truncated domains were isolated from BL21(DE3). A total of 50 ng of CAK was used for in vitro phosphorylation assay. (**E**) Structure of OGFOD1 loop region (239 glycine to 264 glutamate). OGFOD1 structure was modified using PyMOL from PDB ID: 4NHX; loop region (red) and serine 256 (yellow). (**F**) Radioactive kinase assay against His_6_-OGFOD1 WT and S256A. (**G**) In vivo phosphorylation levels. Wild-type or S256A OGFOD1 was overexpressed in OGFOD1 KO MDA-MB-231 cell line. Immunoprecipitation was conducted using an anti-Flag antibody and immunoblotting was accompanied with customized phosphorylated serine 256 antibody. (**H**) In vivo effect of CDK7 and CDK9 on OGFOD1 phosphorylation. Lentiviral knockdown was conducted in MDA-MB-231 cell line. Phosphorylation of OGFOD1 level was confirmed by customized phosphorylated serine 256 antibody. Immunoprecipitation was established using an anti-Flag antibody.

CDK7 and CDK9 phosphorylate CTD serine 5 and serine 2 of RNA polymerase II during initiation and elongation, respectively. Moreover, triple-negative breast cancer cells are reported to be highly addicted to CDK7-dependent transcription, implying the inhibition of CDK7 as a therapeutic target [17]. Using IPs, we evaluated the interconnection between CDKs and OGFOD1. OGFOD1 bound to each component of the CDK7 complex (CAK, CDK7/Cyclin H/MNAT1) and CDK9 complex (P-TEFb, CDK9/Cyclin T) (Figure 5A and Figure S3). We then used an in vitro kinase assay to determine that both recombinant CDKs phosphorylate OGFOD1 in vitro (Figure 5B and Supplementary Figure S3). We designed truncated OGFOD1 constructs according to their functional domains to validate which residue is phosphorylated (Figure 5C). In vitro experiments indicated that phosphorylation levels were elevated in the presence of a loop region (residues 239–264) (Figure 5D). This loop sequence includes a typical CDK substrate motif (PXpS/TP) (Figure 5E), and we substituted serine 256 with alanine to elucidate if this serine is a phosphorylation site. In comparison with wild-type protein, the S256A substitution diminished phosphorylation (Figure 5F and Figure S3). Next, we tested whether serine 256 phosphorylation is detected at the cellular level via customized anti-phosphorylated serine 256 OGFOD1 antibodies and confirmed the specificity (Figure S4). Immunoprecipitation demonstrated that phosphorylation was detected on wild-type OGFOD1 but not the S256A mutant (Figure 5G), and indicated that serine 256 of OGFOD1 was phosphorylated at the cellular level. Subsequently, we tested whether both CDK7 and CDK9 phosphorylated OGFOD1 in vivo. Lentiviral knockdown experiments showed that CDK7 and CDK9 had a functional effect on phosphorylation levels (Figure 5H). Collectively, we found that both CDK7 and CDK9 could phosphorylate serine 256 of OGFOD1 in vitro and in vivo.

### 3.7. CDK7/9 Enhance OGFOD1 Function

**Figure 6:**
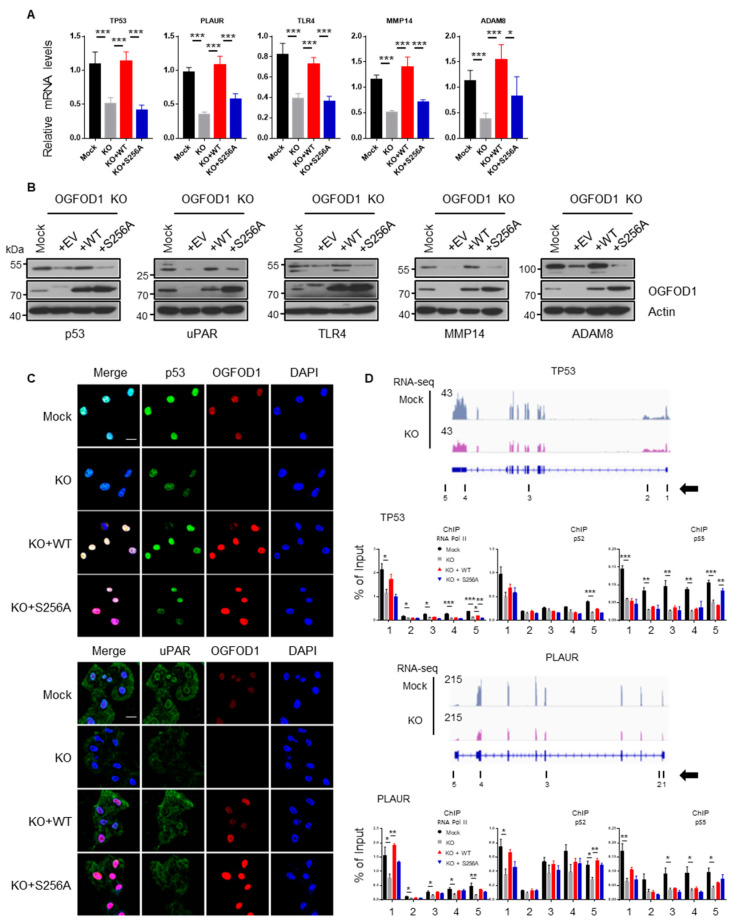
Analysis of genes affected by OGFOD1-knockout. (**A**) Relative mRNA levels in wild-type or mutant (S256A) backup cell lines. Statistical data are presented as mean ± SD. Two-tailed unpaired Student’s t-test was conducted for statistical analysis (*n* = 5) (* *p* < 0.05, ** *p* < 0.01, *** *p* < 0.001). (**B**) Protein levels of downregulated genes in OGFOD1 backup cell lines. (**C**) Immunofluorescence images of target genes. Target proteins were stained with indicated antibodies and Alexa 488 (green). OGFOD1 was stained with Alexa 568 (red), and nuclei were stained with DAPI (blue); scale bar indicates 20 μm. (**D**) Chromatin immunoprecipitation assay on target genes using the indicated antibodies to determine if RNA polymerase II accumulation was affected following OGFOD1 KO or phosphorylation. Measured sites are indicated with black bars and numbers. Arrow indicates gene orientation.

The restoration of wild-type OGFOD1 expression in the OGFOD1 KO cell line significantly rescued the reduced RNA levels; however, the expression of S256A OGFOD1 showed decreased rescue levels of RNA and protein (Figure 6A–C, Figure S5). It indicated that non-phosphorylated S256A OGFOD1 has a weaker ability to enhance transcription. Next, we conducted chromatin IP assay to confirm whether RNA polymerase II accumulation was changed on the target genes. We observed that RNA polymerase II was detached from target genes when OGFOD1 was absent or non-phosphorylated (Figure 6D). This indicates that OGFOD1, cooperating with RNA polymerase II, enhances the expression of genes involved in metastasis, immune response, and receptor signaling in MDA-MB-231 cells. Moreover, CDK7- and/or CDK9-mediated phosphorylation of OGFOD1 on serine 256 was essential for OGFOD1-mediated activation of RNA polymerase II.

### 3.8. Non-Phosphorylated Mutation of Serine 256 Hampers Oncogenic Ability

**Figure 7:**
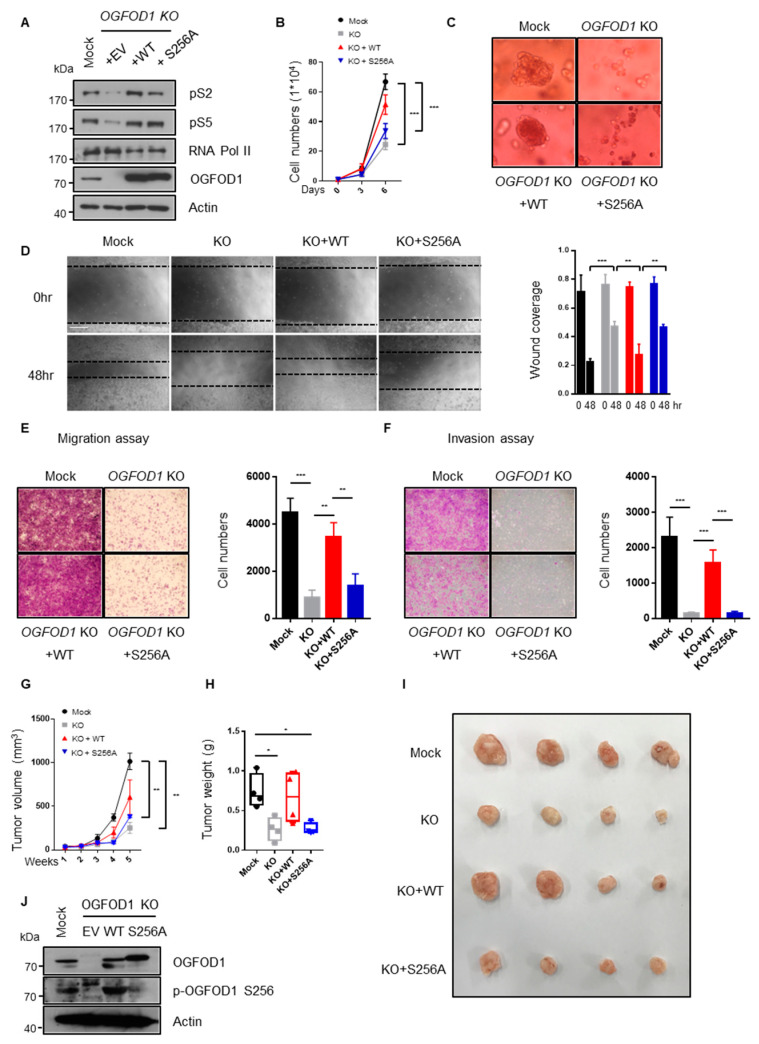
Serine 256 of OGFOD1 has a pivotal role in oncogenic properties. (**A**) Protein levels of OGFOD1 backup in OGFOD1 KO MDA-MB-231 cell lines. (**B**) Proliferation rate of each of the cell lines. Statistical data are presented as mean ± SD. Two-tailed unpaired Student’s t-test was conducted for statistical analysis (*n* = 4) (*** *p*-value < 0.001). (**C**) Three-dimensional cultivation. Cells were embedded on Matrigel and cultivated for 10 days. (**D**) Wound-healing assay. Phase contrast images show the area covered with assigned cells at indicated time points; scale bar indicates 0.3 mm. Statistical data are presented as mean ± SD. Two-tailed one-way ANOVA was conducted for statistical analysis (*n* = 3) (** *p*-value < 0.01, *** *p*-value < 0.001). (**E**,**F**) Transwell migration and invasion assay. Migrating and invading cells were stained with crystal violet and measured. Statistical data are presented as mean ± SD. Two-tailed one-way ANOVA was conducted for statistical analysis (*n* = 3,4) (** *p*-value < 0.01, *** *p*-value < 0.001). (**G**–**I**) In vivo function of OGFOD1. Assigned groups (mock, KO, KO + WT, and KO + S256A) were subcutaneously injected into mice. Tumor volumes were measured every week. Statistical data are presented as mean ± SEM. Two-tailed one-way ANOVA was conducted for statistical analysis (*n* = 4) (** *p*-value < 0.01). Tumor weights were measured after the mice were euthanized. Statistical data are presented as minimum to maximum with all points. Two-tailed one-way ANOVA was conducted for statistical analysis (*n* = 4) (* *p*-value < 0.05). (**J**) OGFOD1 expression was confirmed in tumor tissue samples.

We expressed wild-type or non-phosphorylated S256A OGFOD1 in the OGFOD1 KO cell line to investigate the physiological significance of S256 phosphorylation in oncogenic properties. S256A OGFOD1 failed to restore pS2 levels (Figure 7A). The expression of wild-type OGFOD1 almost restored the proliferation rate, although restoration of S256A-mutated OGFOD1 expression abrogated rapid proliferation (Figure 7B). Cancer growth is affected by not only the proliferation rate but also by the extracellular environment and structure. In a three-dimensional embedded culture on Matrigel, OGFOD1 KO cells had significantly decreased growth; however, the expression of wild-type OGFOD1 restored the growth to a similar level as that seen in mock cells. By contrast, the expression of S256A-OGFOD1 failed to grow in an anchorage-independent manner (Figure 7C). Similarly, OGFOD1 KO- and S256A-OGFOD1-expressing cells significantly reduced migration and invasion, which are distinct characters of cancer (Figure 7D–F). Thus, these results support the contribution of the phosphorylation state of S256 to ongoing tumor development in MDA-MB-231 cells. Subsequently, to evaluate in vivo functions, we injected MDA-MB-231 cells subcutaneously into mice. Xenograft tumor experiments were assigned to four groups: control, OGFOD1 KO, KO + WT, and KO + S256A (Figure 7G–J). With OGFOD1 deletion, tumor growth was remarkably inhibited in agreement with what we observed in vitro. Notably, the restoration of wild-type OGFOD1 expression rescued tumor growth; however, the expression of the S256A OGFOD1 abated the oncogenic effect.

## 4. Discussion

Although several studies have attempted to examine the physiological role of OGFOD1, the exact action of OGFOD1 in cancer remains undescribed. Recent reports have detailed a high expression level of OGFOD1 in proportion to cancer progression induced by oncogenes or microRNAs that are associated with poor prognosis in tumors, including chronic lymphocytic leukemia, breast cancer, laryngeal papilloma, and colon cancer [7,8,9,10]. Despite evidence that OGFOD1 promotes tumorigenesis, the precise mechanism is not well understood.

Here, we showed that loss of OGFOD1 reduced RNA polymerase II transcriptional activity in cancer, leading to a reduction in metastatic gene expression and tumor growth retardation, and that this effect was related to the nuclear position of OGFOD1.

The stepwise activation of RNA polymerase II-mediated transcription is due to the CDK7 (CAK) and CDK9 (P-TEFb) complexes having distinct substrates. Shortly after transcription initiation, RNA polymerase II travels a few nucleotides from the transcription start site and halts transcription. This phenomenon, called “Pausing”, is conducted by 5,6-dichloro-1-beta-D-ribofuranosylbenzimidazole sensitivity-inducing factor (DSIF) [26] and NELF [27] and leads to the accumulation of RNA polymerase II at the promoter proximal region. To continue to transcribe and fully enter the elongation cycle, P-TEFb subsequently phosphorylates DSIF and NELF, which are then released from RNA polymerase II [28,29]; CDK7 is known to be required for activating CDK9 phosphorylation and CDK9-dependent downstream events [30].

Here, we showed that CDK7 and CDK9 both phosphorylate serine 256 on OGFOD1 in vitro (Figure 5 and Figure S3). CDK7 knockdown in MDA-MB-231 cells reduced the level of phosphorylated OGFOD1 (S256) to a more significant degree than knockdown of CDK9 did (Figure 5H), suggesting that CDK7 may be a preferential kinase for OGFOD1 (S256) in vivo. However, the knockdown efficiencies were little different, and S256A OGFOD1 could rescue pS5 levels as much as wild-type OGFOD1 (Figure 7A). Moreover, Spt5, a component of DSIF, is phosphorylated by both kinases in vitro [31,32]. Similarly, c-MYC associates with and recruits P-TEFb to the promoter while facilitating CDK7-dependent assembly of Spt5–RNA polymerase II complexes [33,34]. Therefore, we could not exclude which kinase preferentially affected the function of OGFOD1 in RNA polymerase II-mediated transcription in vivo.

At this stage, how OGFOD1 selects the target genes remains unclear because the deletion of OGFOD1 reduced the transcription of both oncogenic and tumor suppressor genes (Supplementary Table S2). This implies that OGFOD1 may participate in the activation of genes following their environmental conditions through promoting RNA polymerase II transcriptional activity in a cellular context-dependent manner. TP53 is a well-described tumor suppressor gene; however, TP53 in MDA-MB-231 cells is mutated to TP53(R280K), which is also oncogenic [35,36]. Thus, the reduction in oncogenic p53(R280K) expression in OGFOD1-knockout MDA-MB-231 cells interferes with tumor development in this context.

Urokinase-type plasminogen activator receptor, uPAR, an OGFOD1 target gene, recruits uPA on the cell membrane to trigger extracellular matrix degradation, which is a frequently upregulated pathway in cancers [37]. In agreement with this signaling function, uPAR promotes epithelial–mesenchymal transition [38], whereas blocking uPAR function in breast cancer exhibits a diminished cancer profile [39,40]. This indicates that reduced uPAR function following OGFOD1 knockout prevented tumor progression. Likewise, decreased levels of metalloproteinase MMP9 and ADAM8 also shrank tumor progression on this basis [41,42]. Toll-like receptor (TLR) functions as a key regulator of innate immune response by recognizing pathogens [43]. Lately, an additional role of TLR in cancer has been reported. TLR4 is highly expressed in MDA-MB-231 cells [44], which have enhanced tumorigenesis potential, and the inhibition of TLR4 inhibited this process [45,46,47]. Collectively, in MDA-MB-231 cells, OGFOD1 knockout decreased several metastasis-associated genes, resulting in the inhibition of cancer development.

Recently, several groups have reported that many cancers, such as triple-negative breast cancer, ovarian cancer, thyroid cancer, and uveal melanoma, exhibit CDK-dependent transcription addiction [16,17,18,19,48]. OGFOD1 also has an oncogenic property in these contexts and phosphorylation of OGFOD1 by CDKs stimulates tumor formation; therefore, we suggest that there is an overlapping role in tumorigenesis. Indeed, non-phosphorylated substitution hampered OGFOD1 oncogenic characteristics, including rapid proliferation, invasion, and metastasis (Figure 7), implying that CDK-mediated phosphorylation of OGFOD1, as well as RNA polymerase II, endows aggressive tumor proliferation. These phenomena could additionally explain how cancer is addicted in transcription.

## 5. Conclusions

Despite the rational evidence between OGFOD1 and cancer, the exact relationship is poorly understood. Here, we observed that the elimination of OGFOD1 causes a reduction in tumor improvement. We demonstrated that OGFOD1 directly interacts with the C-terminal domain of RNA polymerase II, regulating metastatic gene expression in their context by altering CTD phosphorylation levels. Consequently, we identified that OGFOD1 is associated with tumor progress through enhancing RNA polymerase II-dependent transcription in MDA-MB-231. In addition, CDK7/9 phosphorylate serine 256 of OGFOD1, which promotes its driving ability in cancer both in vitro and in vivo.

## Data Availability

The data presented in this study are available from the corresponding author.

## Supplementary Materials

The following are available online at https://www.mdpi.com/article/10.3390/cancers13143418/s1, Figure S1: Knockout of OGFOD1 in MDA-MB-231 by CRISPRCas9 technology, Figure S2: Altered genes by OGFOD1 KO in MDA-MB-231, Figure S3: P-TEFb phosphorylates serine 256 residue of OGFOD1 in vitro, Figure S4: The generation of customized anti-phosphorylated serine 256 OGFOD1 antibody, Supplementary Figure S5: Altered gene expression levels in Mock, OGFOD1 KO, KO+WT, KO+S256A, Figures S6–S15: Source data, Table S1: Primer list for knockout, RT-qPCR, and ChIP-qPCR, Table S2: RNA sequencing data values for mock versus OGFOD1 knockout.
