# Supplementary material for: Phosphorylation of OGFOD1 by Cell Cycle-Dependent Kinase 7/9 Enhances the Transcriptional Activity of RNA Polymerase II in Breast Cancer Cells
Source: Cancers (Basel). 2021 Jul 8;13(14):3418. doi: 10.3390/cancers13143418 (PMC8304009; doi:10.3390/cancers13143418)
Supplement: Supplementary file 1 [file cancers-13-03418-s001.zip › cancers-1262044-supple-xml.pdf]

cancers

MDPI

Supplementary Materials: Phosphorylation of OGFOD1 by Cell Cycle-Dependent Kinase 7 Enhances the Transcriptional Activity of RNA Polymerase II in Breast Cancer Cells

Han-Teo Lee, Il-Hwan Lee, Jae-Hwan Kim, Sangho Lee, Sojung Kwak, Min Young Suh, In-Young Hwang, Bu-Gyeong Kang, Sun-Shin Cha, Byung Il Lee, Sang Eun Lee, Jinmi Choi, Jae-Seok Roe, Eun-Jung Cho, Hong-Duk Youn

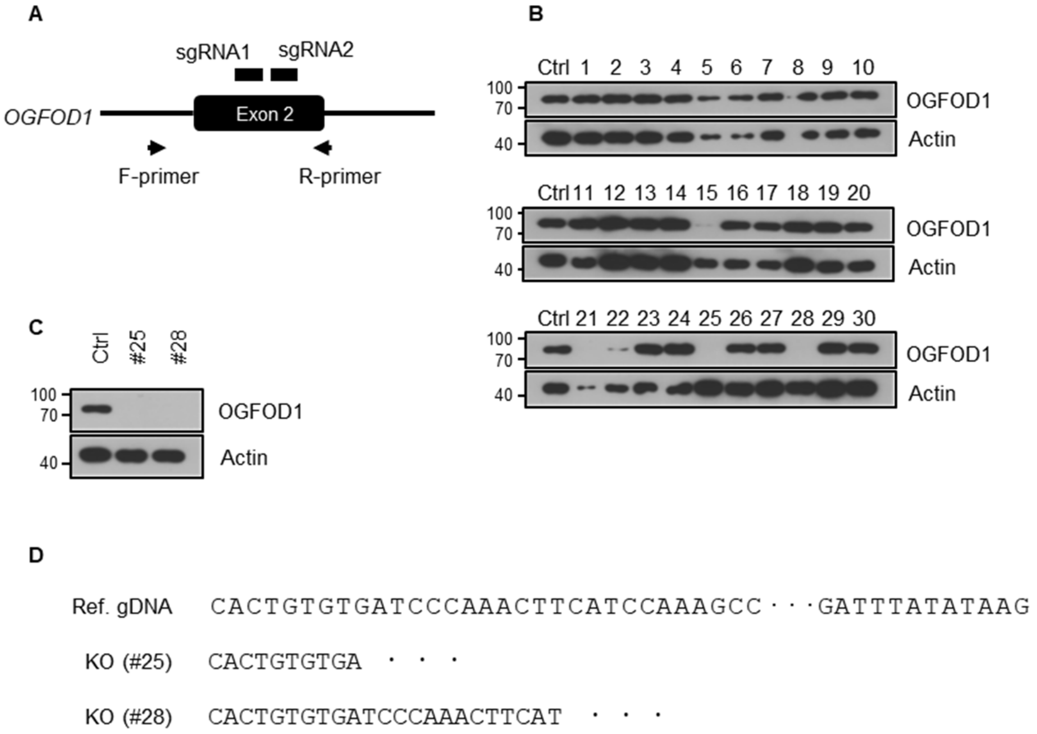

Figure S1. Knockout of OGFOD1 in MDA-MB-231 by CRISPRCas9 technology. (**A**) a model of OGFOD1 knockout strategy. sgRNAs show target sites of guide RNA. Each of sites was digested using nickase Cas9 (D10A) enzyme. F-primer and R-primer are primer sets for DNA amplification to analysis the deletion sequence. (**B**,**C**) The screening results of OGFOD1 knockout. Protein levels were confirmed by western blot assay. Actin were used as control. (**D**) Sanger sequencing results in knockout #25 and #28 cell lines.

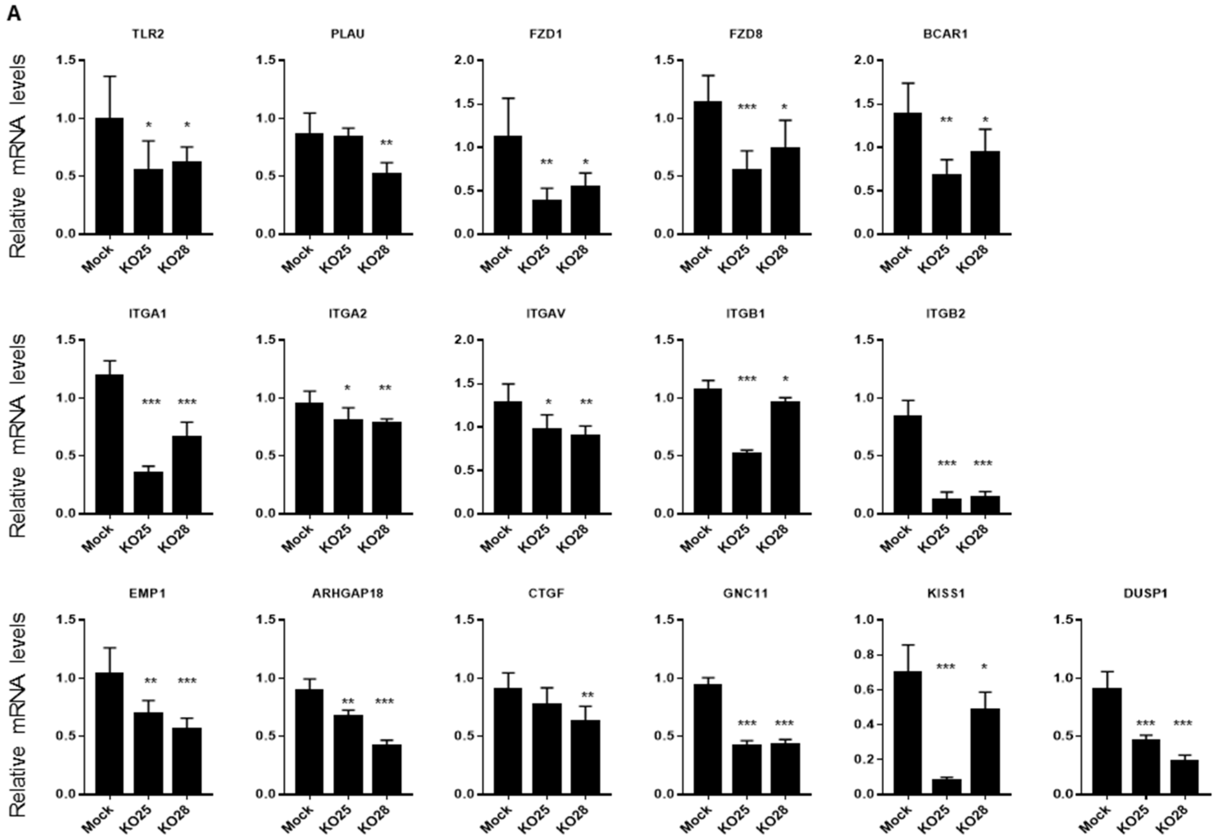

Figure S2. Altered genes by OGFOD1 KO in MDA-MB-231. (**A**) mRNA expression of target genes. Relative mRNA levels were analyzed by qRT-PCR. In comparison with Mock cells, OGFOD1 KO cells exhibits decreased mRNA levels. Statistical data were presented as mean ± SD. Two-tailed unpaired student t-test was performed for statistical analysis. (n = 6), (* $p < 0.05$, ** $p < 0.01$, *** $p < 0.001$).

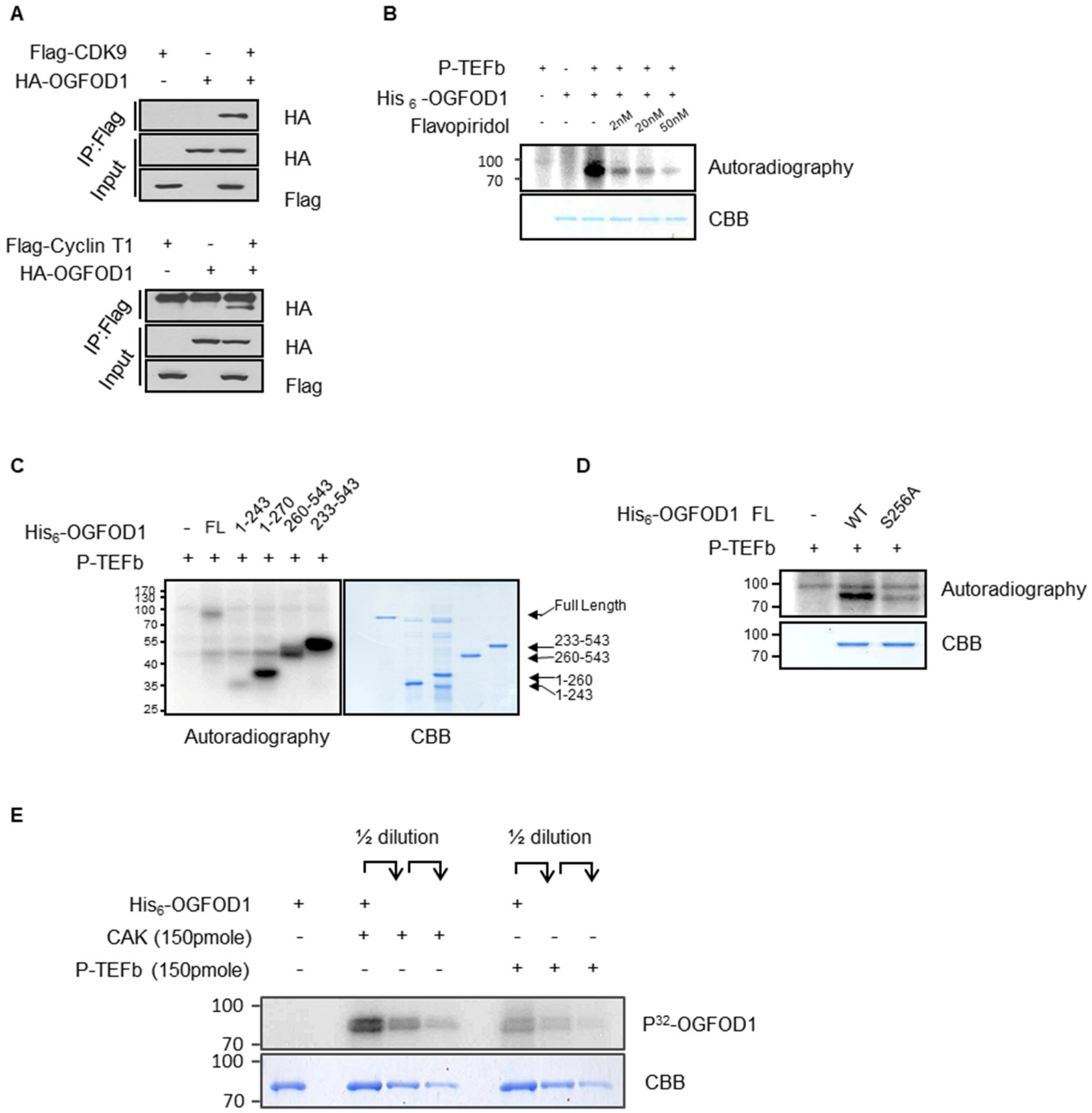

Figure S3. P-TEFb phosphorylates serine 256 residue of OGFOD1 in vitro. (**A**) P-TEFb complex (CDK9, Cyclin T1) was transiently transfected in HEK293T with or without OGFOD1. Immunoprecipitation was performed by anti-Flag. (**B**) *In vitro* P-TEFb phosphorylation assay. Recombinant P-TEFb and OGFOD1 proteins were used. Flavopiridol was used as an inhibitor of kinase assay. OGFOD1 protein amounts were confirmed by coomassie brilliant blue (CBB). (**C**) In vitro P-TEFb phosphorylation assay with truncated OGFOD1. 100ng of P-TEFb was used for in vitro phosphorylation assay. (**D**) Radioactive kinase assay of wild type or S256A OGFOD1. Kinase assay was done with same amount of wild type or mutant OGFOD1. Input proteins were verified with coomassie brilliant blue staining. (**E**) *In vitro* phosphorylation assay of CDK7 and CDK9 with serial dilution of OGFOD1 proteins.

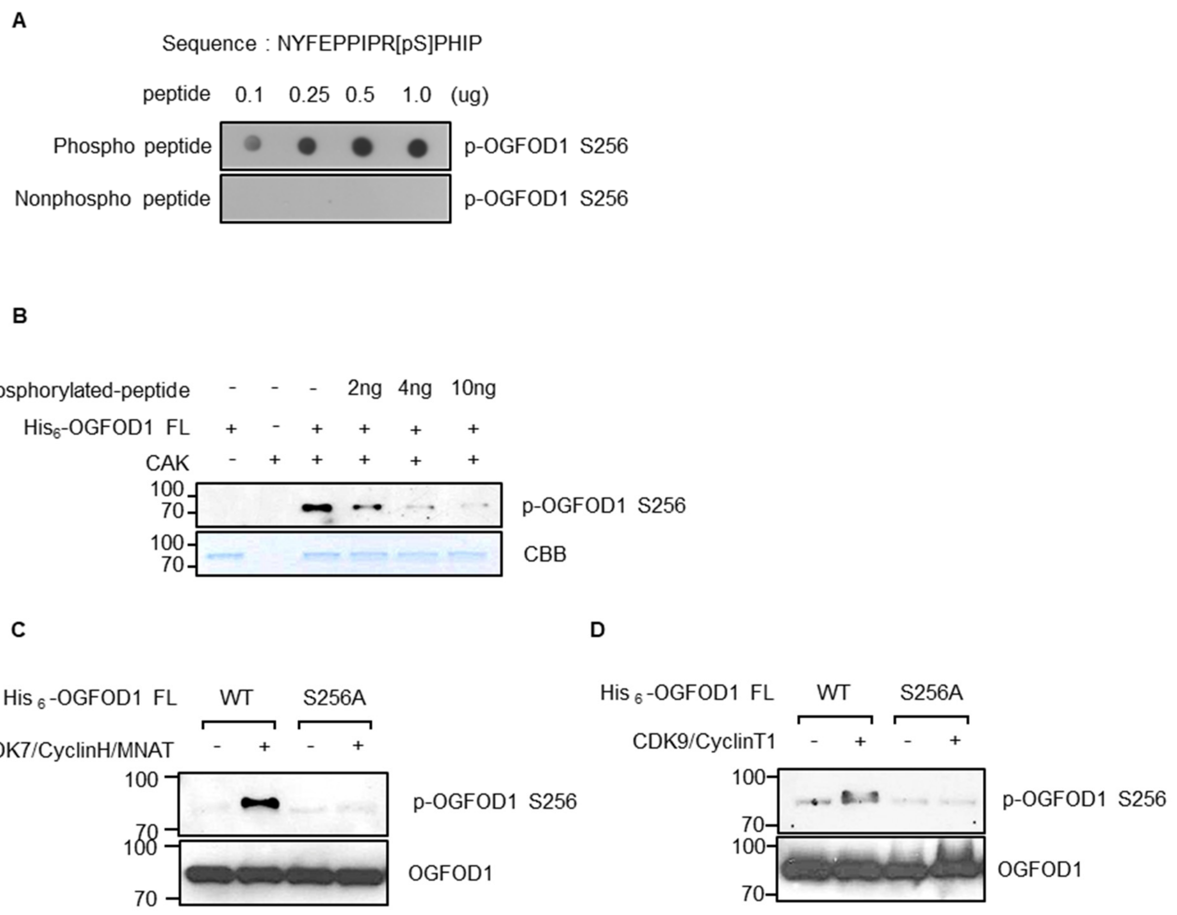

Figure S4. The generation of customized anti-phosphor serine 256 OGFOD1 antibody. (**A**) Dot blot assay. Anti-phosphor serine 256 OGFOD1 antibody was generated against NYFEPPIRP[pS]PHIP. Phosphor or non-phosphor peptide was blotted on nitrocellulose membrane with indicated amounts. Dot blot assay was conducted to confirm its specificity. (**B**) Peptide competition assay. Recombinant OGFOD1 was phosphorylated by CAK and separated by SDS-PAGE. Phosphor peptide was treated with primary antibody. (**C**) *In vitro* CAK phosphorylation. Recombinant OGFOD1 was phosphorylated by CAK complex. Immunoblotting was performed using p-OGFOD1 S256 antibody. (**D**) In vitro P-TEFb phosphorylation. Recombinant OGFOD1 was phosphorylated by CAK complex. Immunoblotting was performed using p-OGFOD1 S256 antibody.

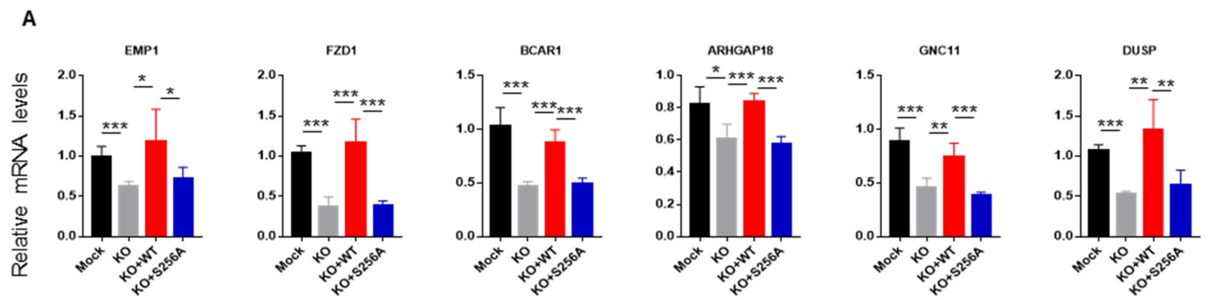

Figure S5. Altered gene expression levels in Mock, OGFOD1 KO, KO+WT, KO+S256A. (**A**) mRNA expression of target genes in OGFOD1 rescued cells. Statistical data were presented as mean ± SD. Two-tailed unpaired student t-test was performed for statistical analysis. (n = 5), (* $p < 0.05$, ** $p < 0.01$, *** $p < 0.001$).

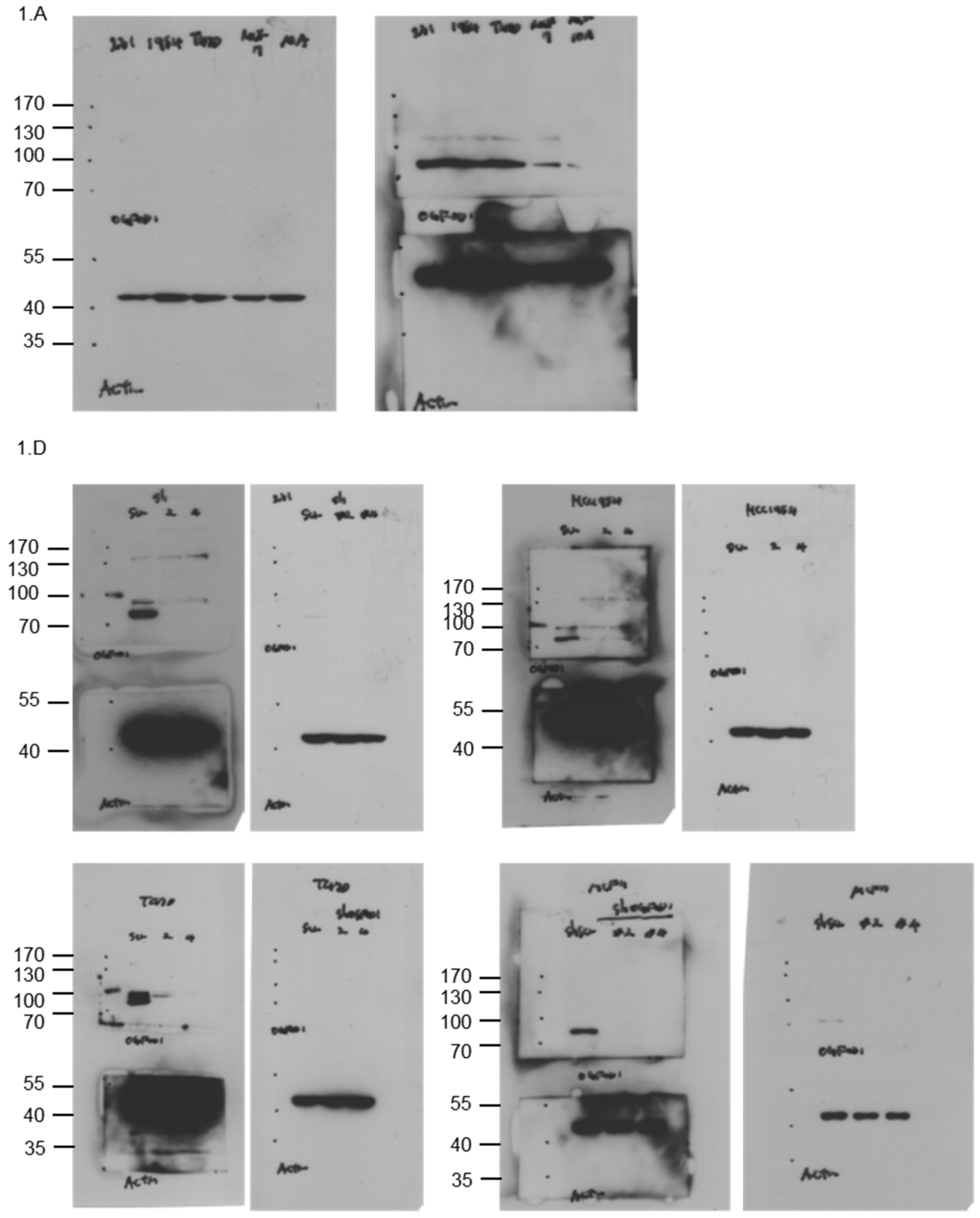

Figure S6. Full western blot of Figure 1.

2.A

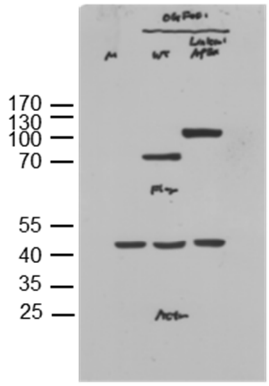
170
130
100
70
55
40
35
25

2.D

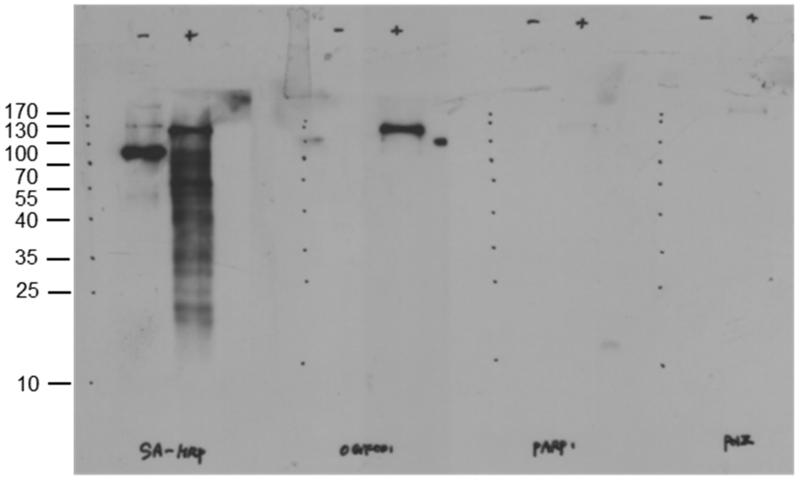
170
130
100
70
55
40
35
25
10

2.E

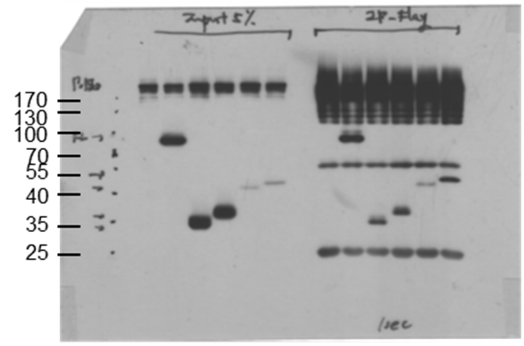
170
130
100
70
55
40
35
25

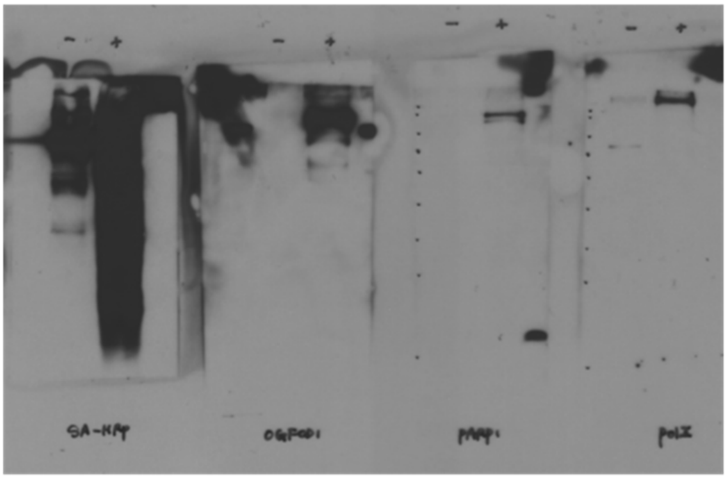

2.F

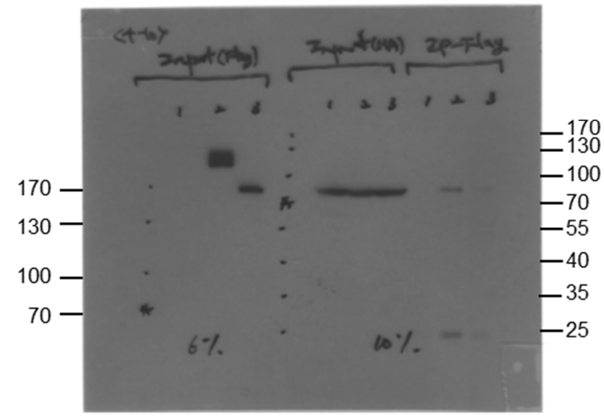
170
130
100
70
170
130
100
70
55
40
35
25

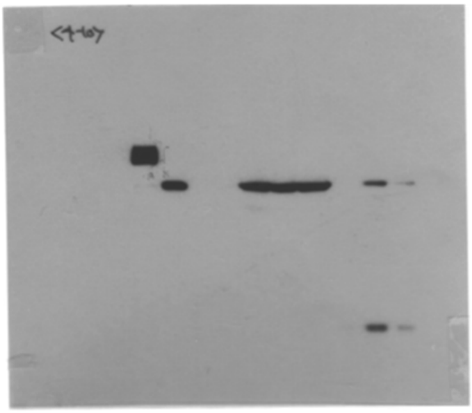

2.G

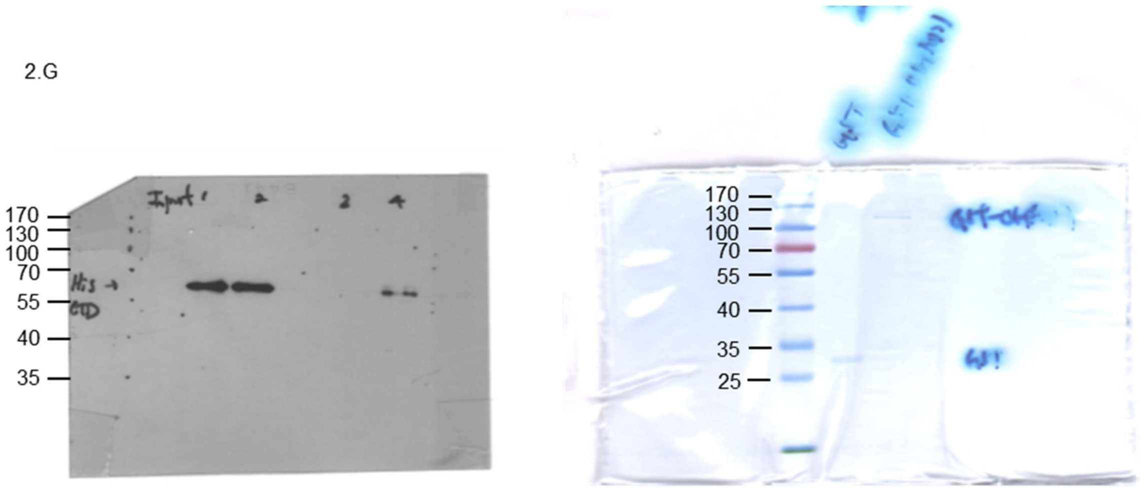

2.H

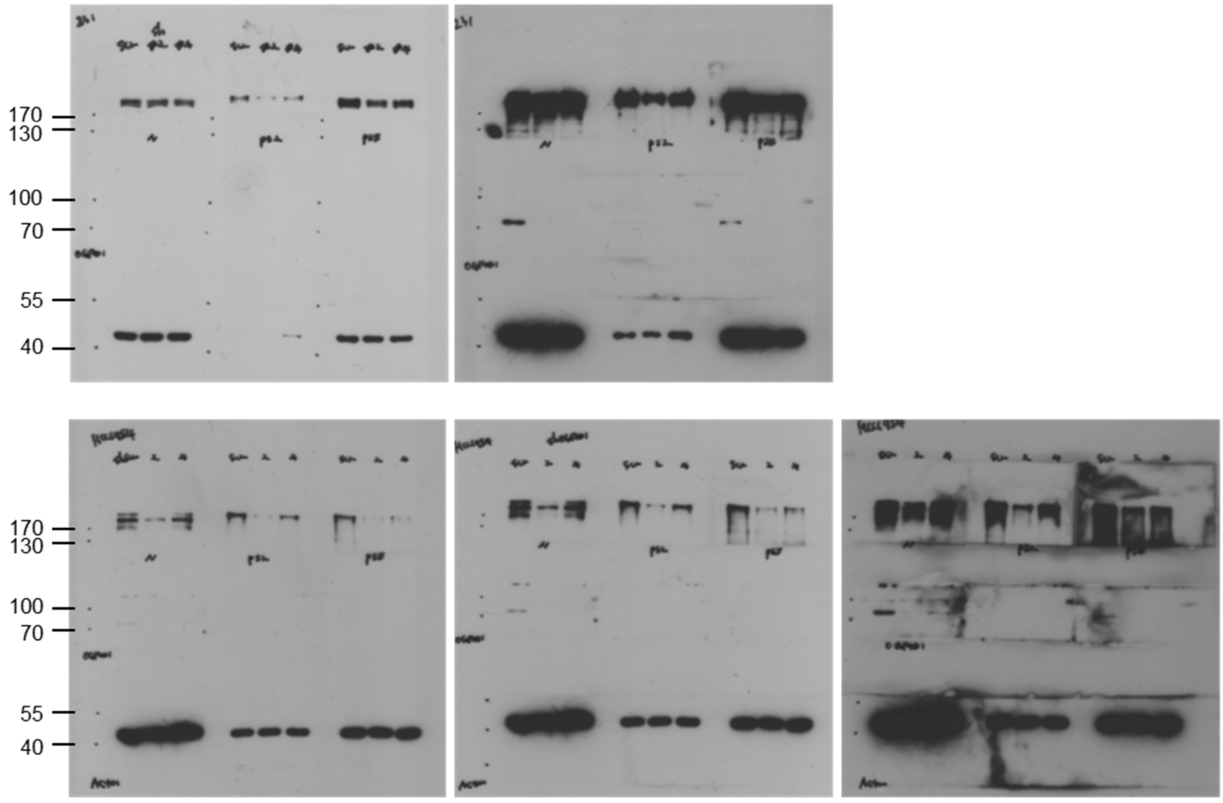

2.H

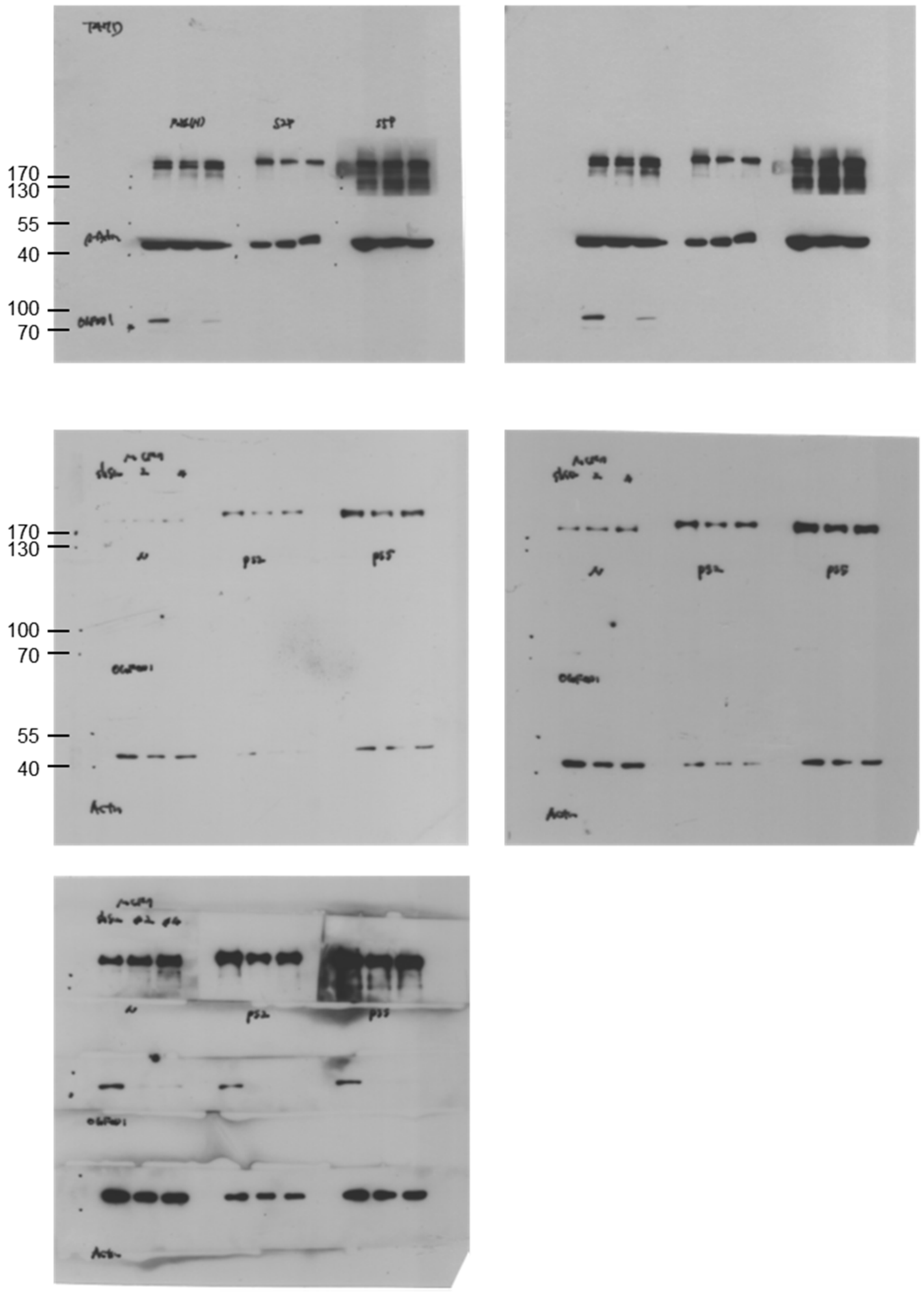

Figure S7. Full western blot of Figure 2.

3.A

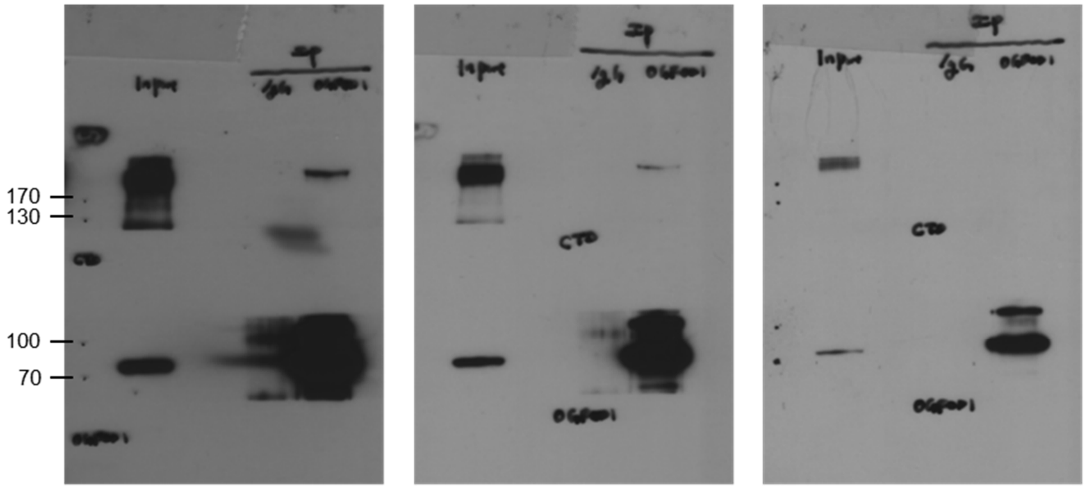
Input
IP
IgG
OGFOD1
CTD
170
130
100
70

3.B

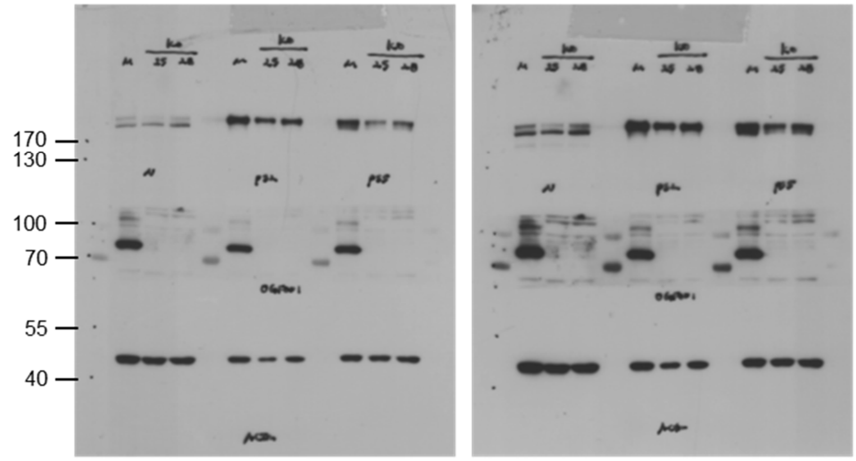
KO
170
130
100
70
55
40

3.D

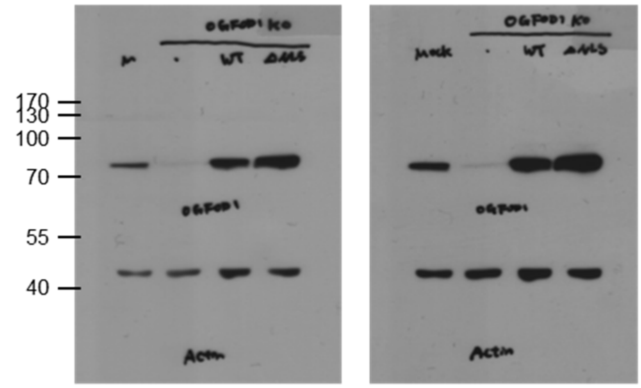
OGFOD1 KO
Mock
WT
170
130
100
70
55
40
Actin

3.F

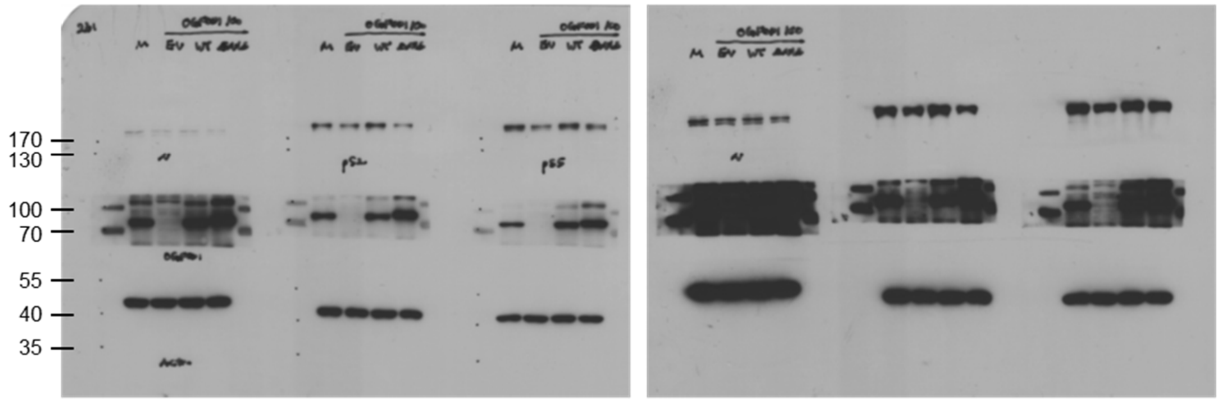

Figure S8. Full western blot of Figure 3.

4.D

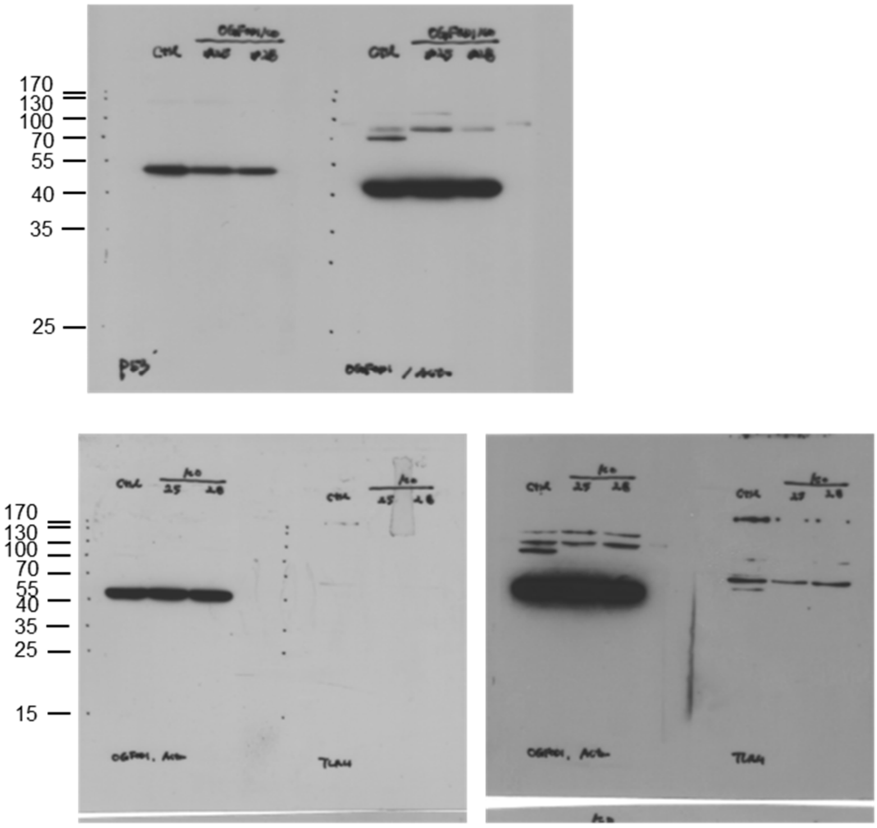

4.D

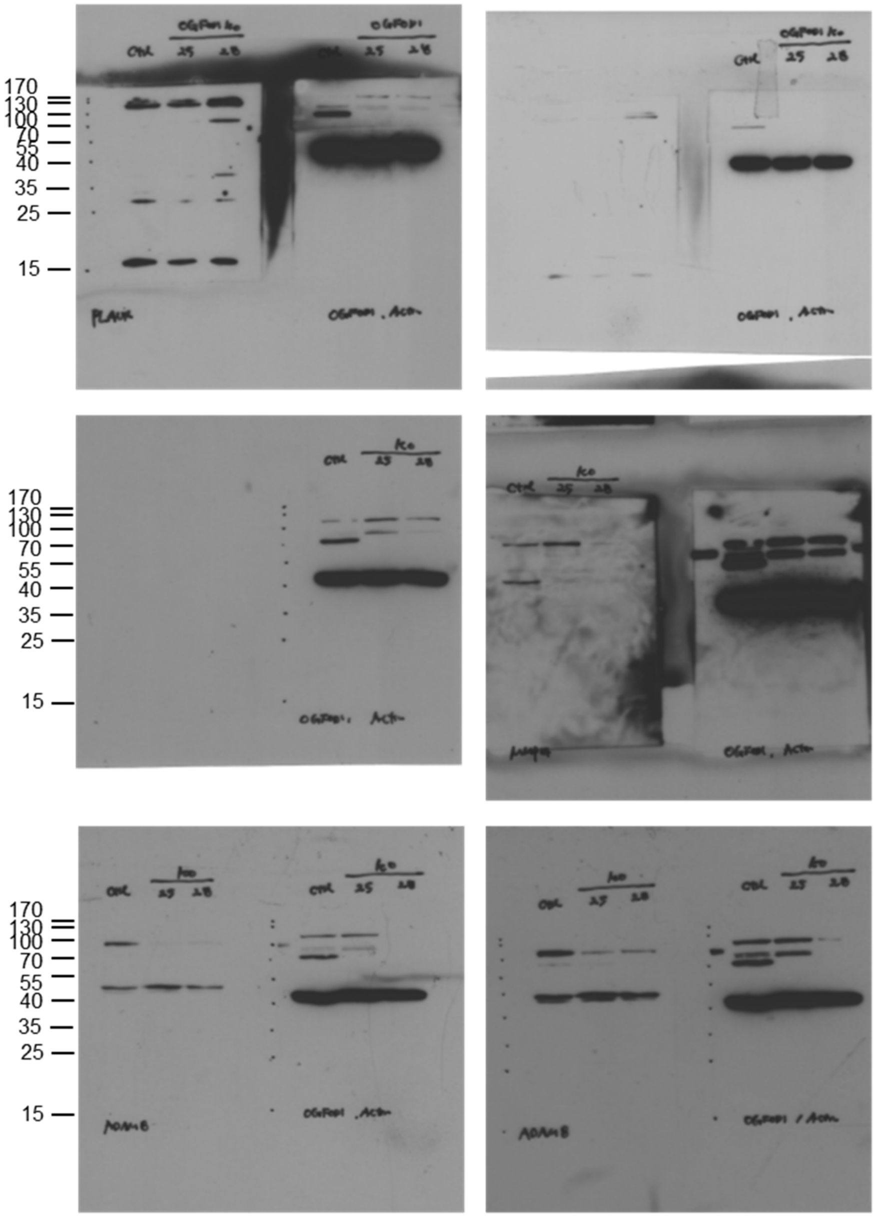

Figure S9. Full western blot of Figure 4.

5.A

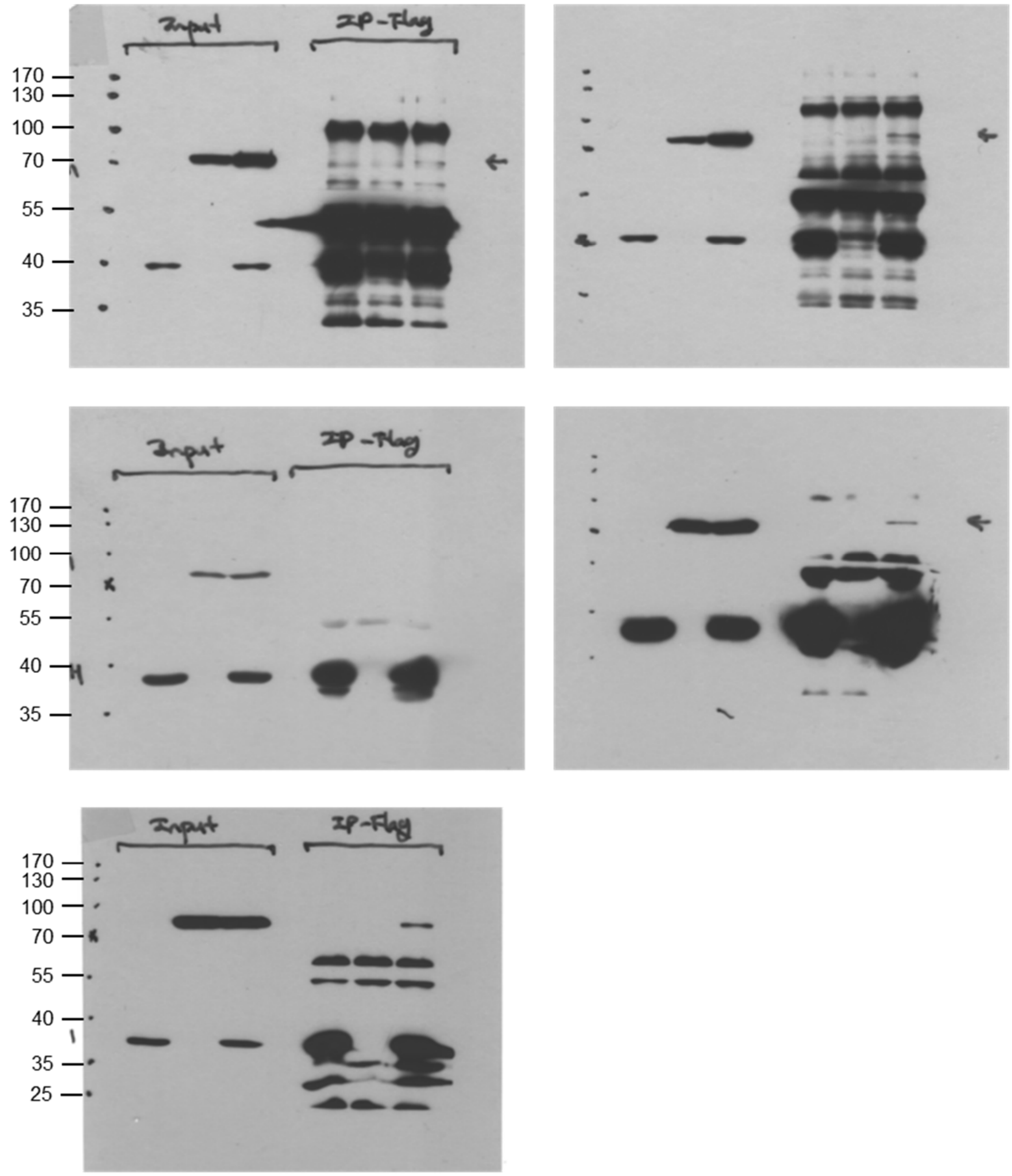
Input
IP-Flag
170
130
100
70
55
40
35
Input
IP-Flag
170
130
100
70
55
40
35
Input
IP-Flag
170
130
100
70
55
40
35
25

5.B

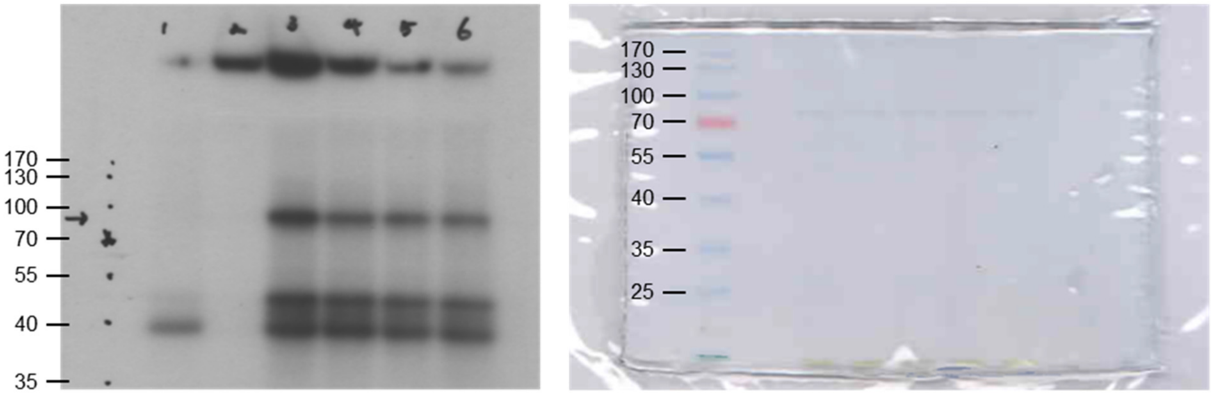
170
130
100
70
55
40
35
170
130
100
70
55
40
35
25

5.D

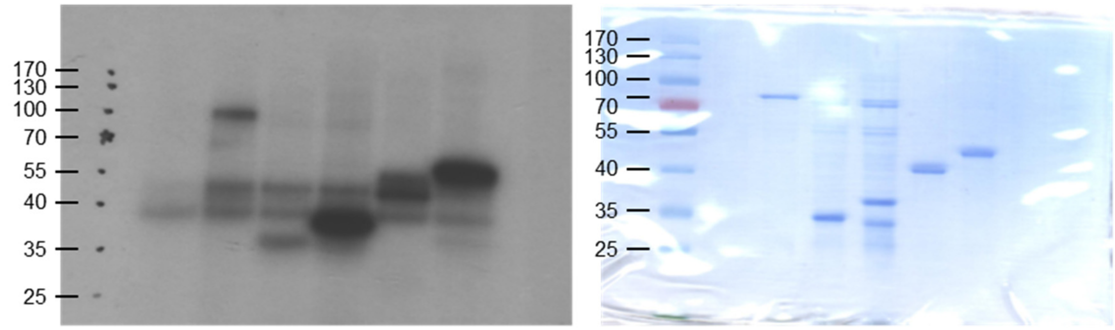
170
130
100
70
55
40
35
25
170
130
100
70
55
40
35
25

5.F

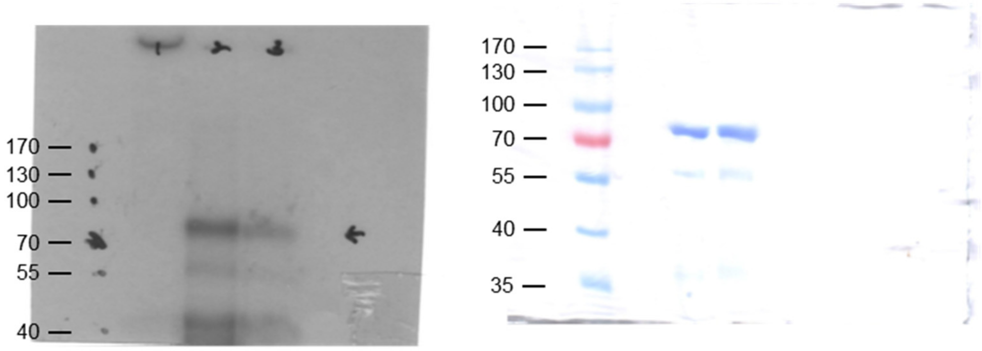
170
130
100
70
55
40
170
130
100
70
55
40
35

5.G

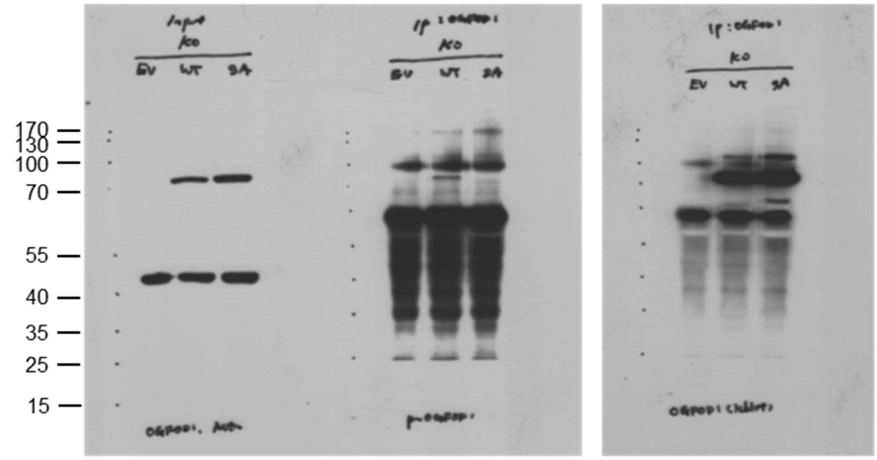

5.H

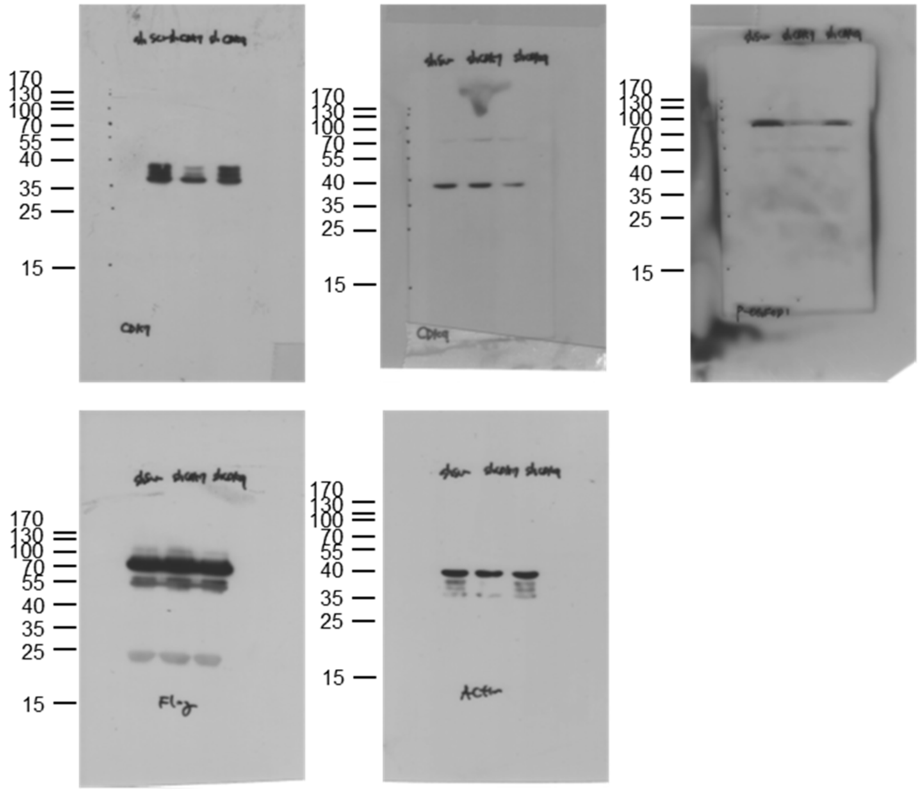

Figure S10. Full western blot of Figure 5.

6.B

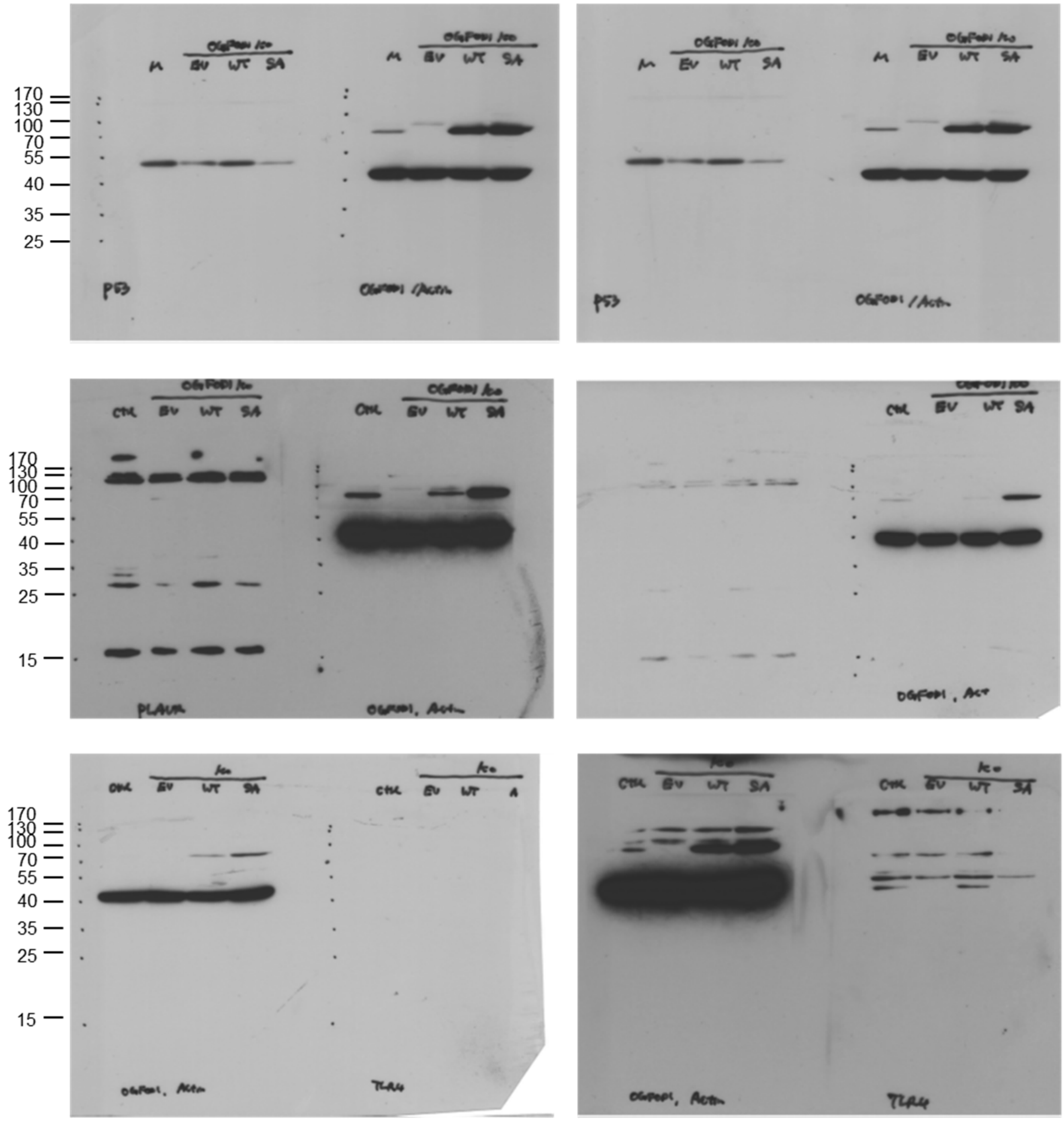

6.B

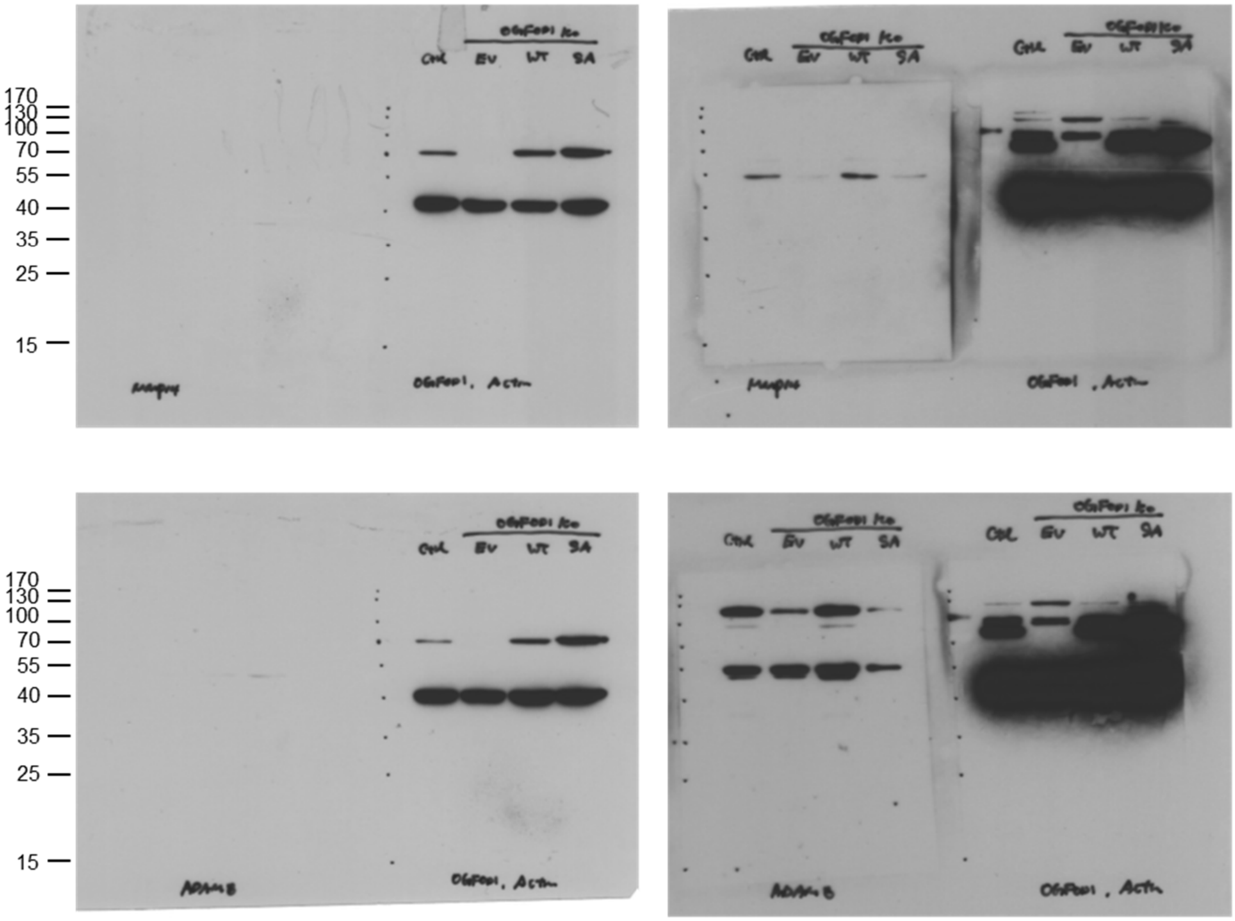

Figure S11. Full western blot of Figure 6.

7.A

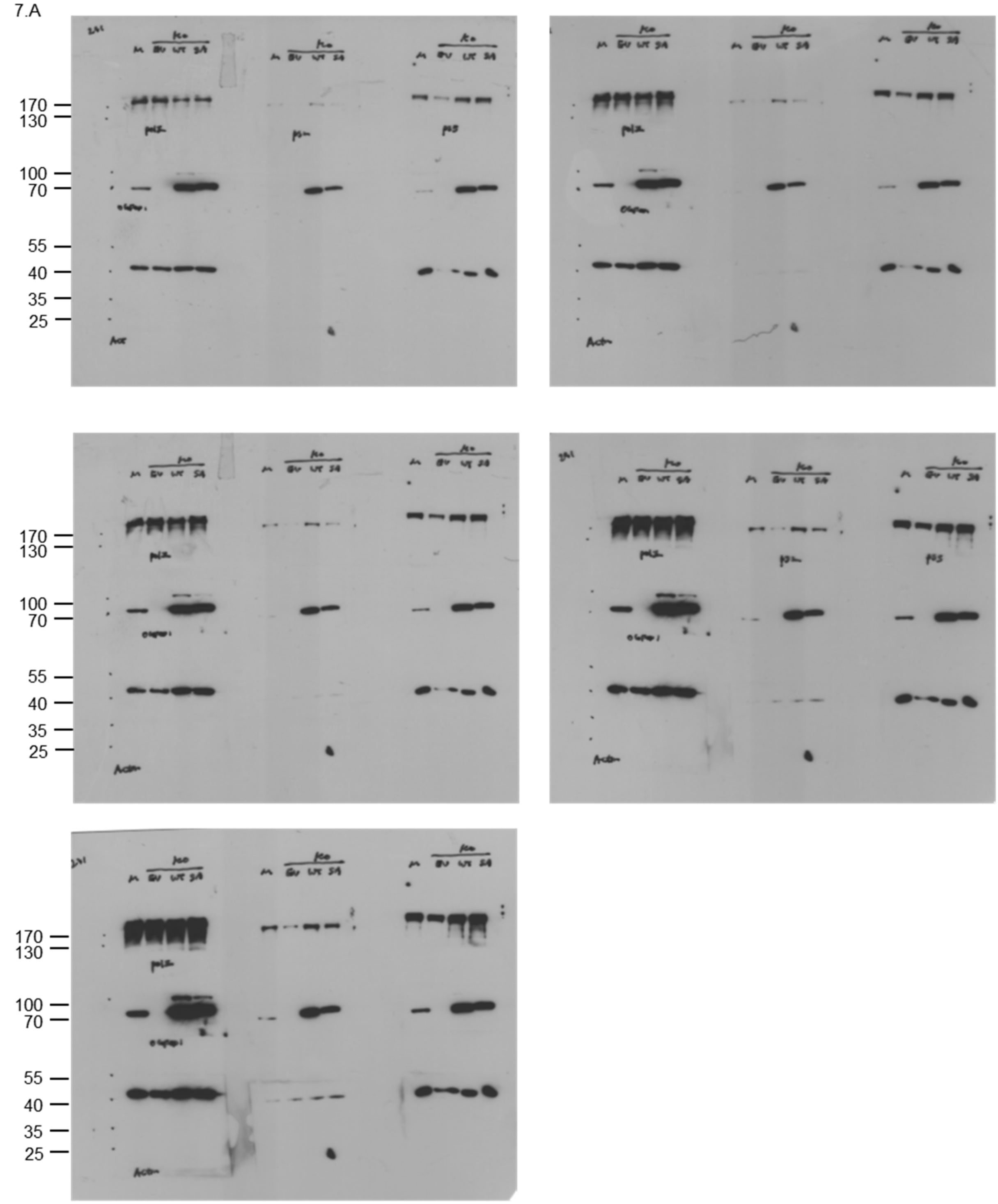

7.J

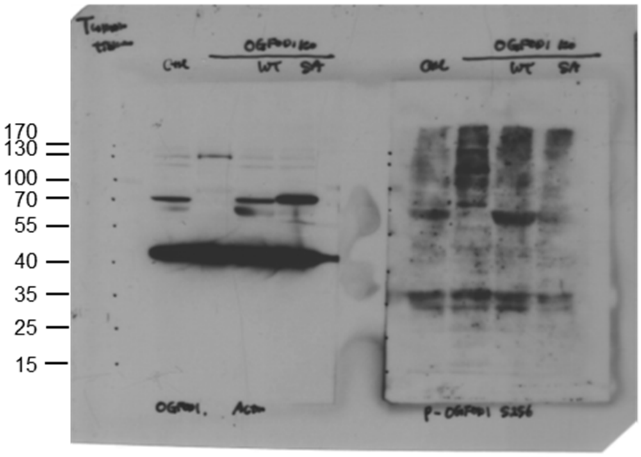

Figure S12. Full western blot of Figure 7.

1.A

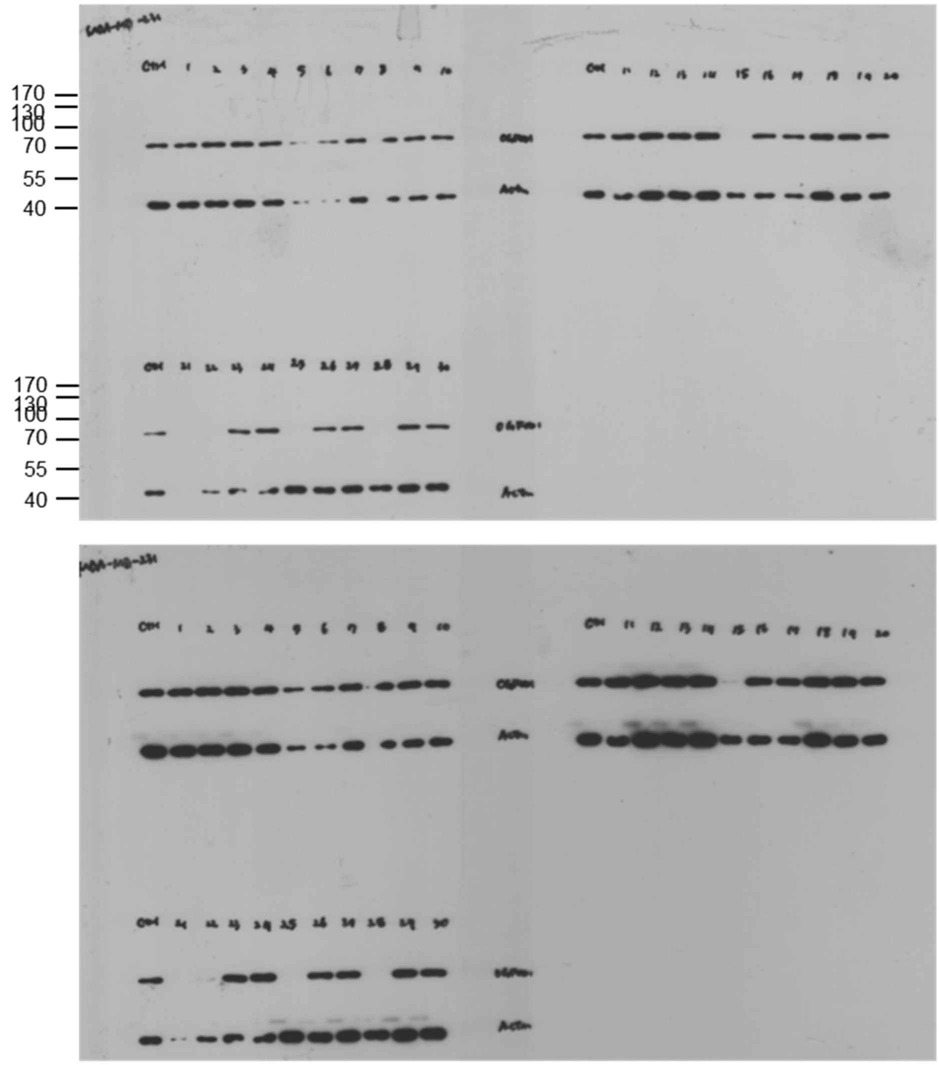

1.C

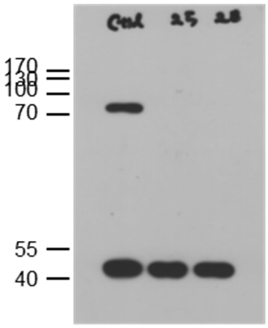

Figure S13. Full western blot of Figure S1.

3.A

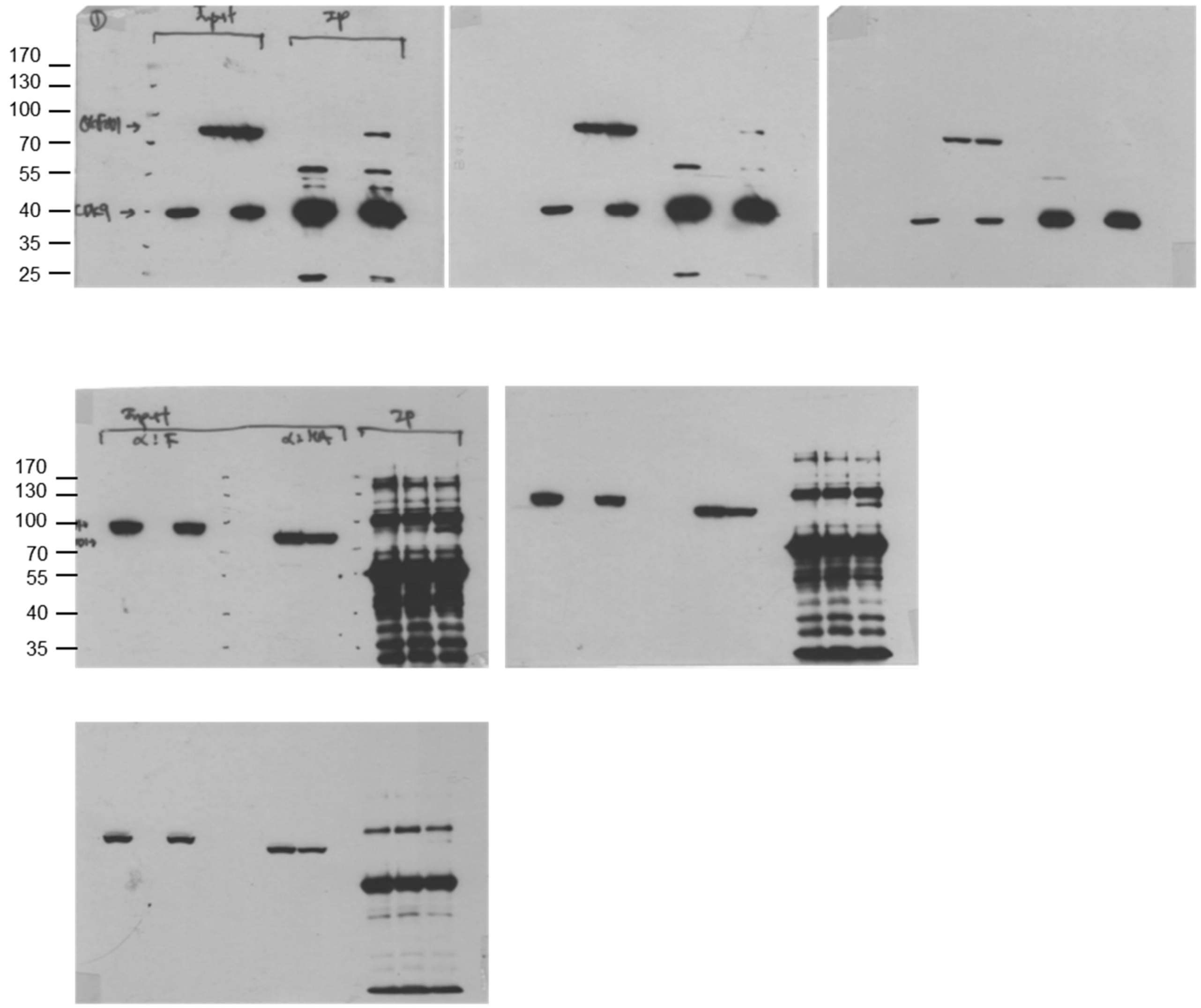
170
130
100
70
55
40
35
25
170
130
100
70
55
40
35

3.B

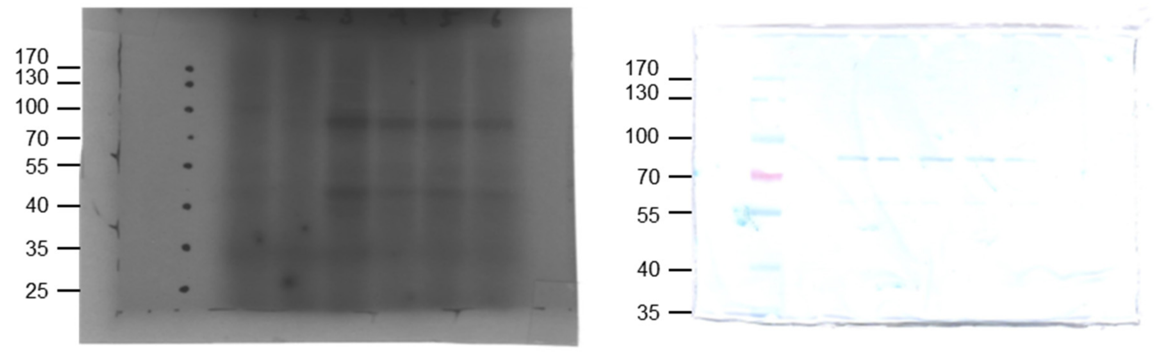
170
130
100
70
55
40
35
25
170
130
100
70
55
40
35

3.C

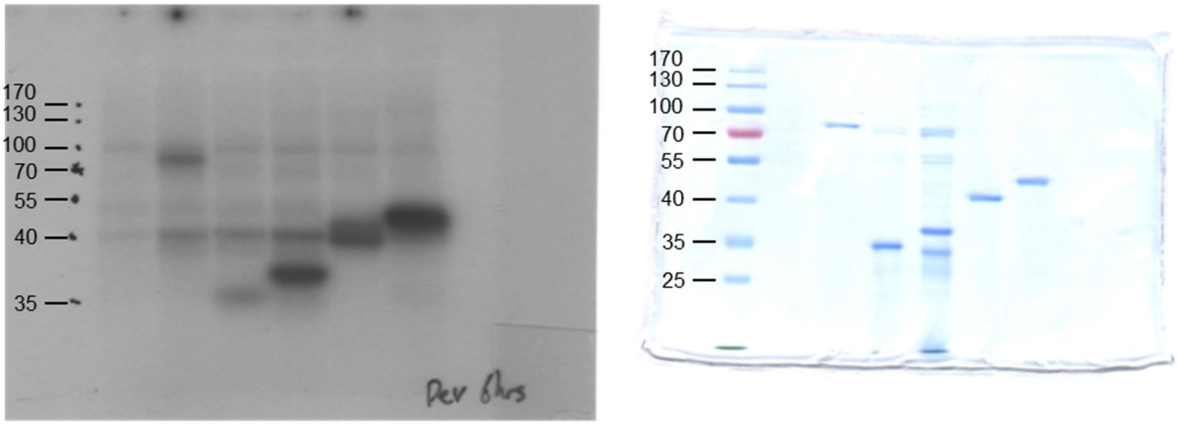

3.D

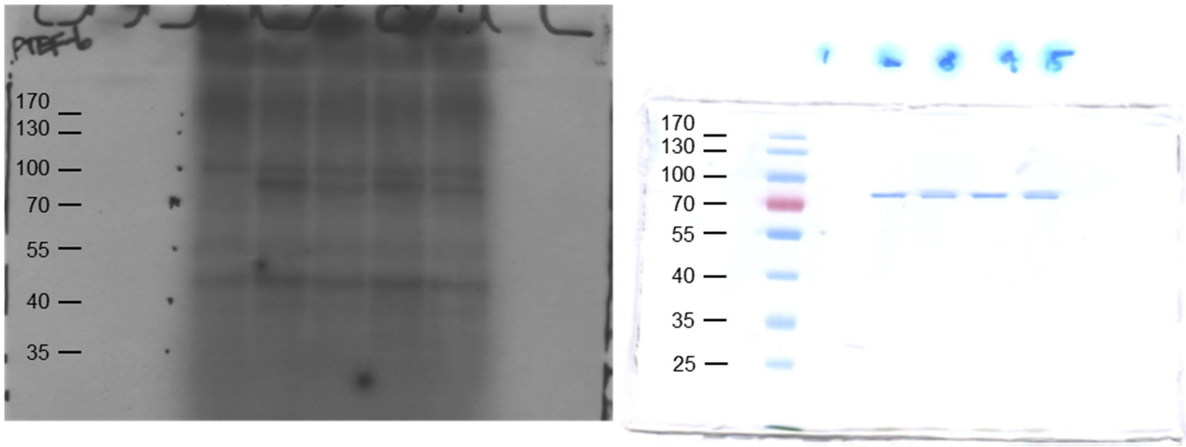

3.E

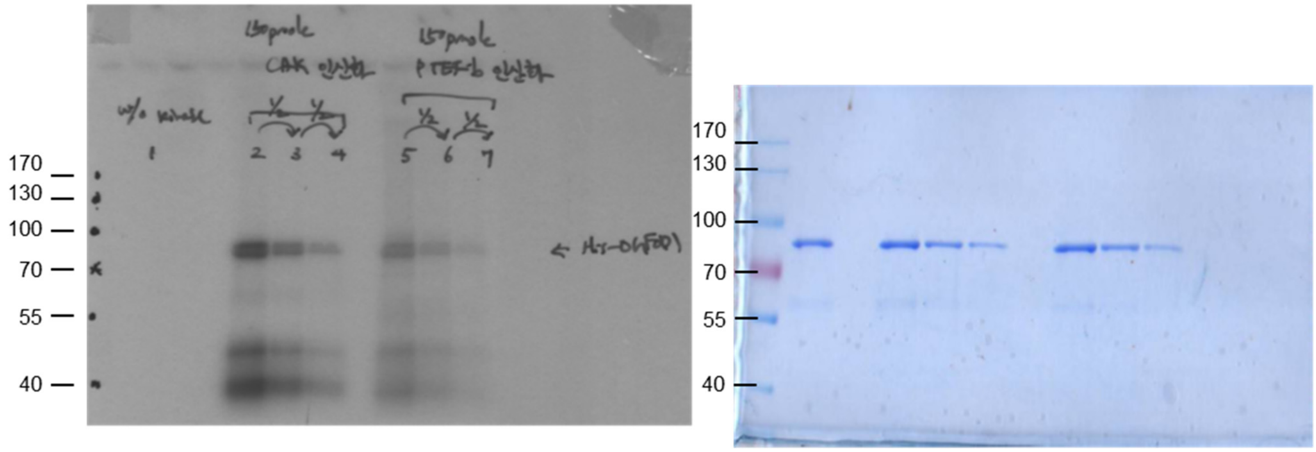

Figure S14. Full western blot of Figure S3.

4.A

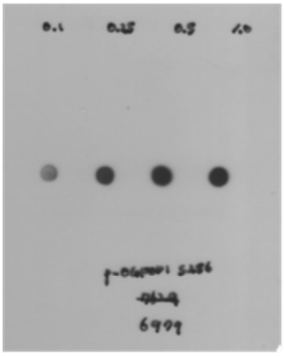

4.B

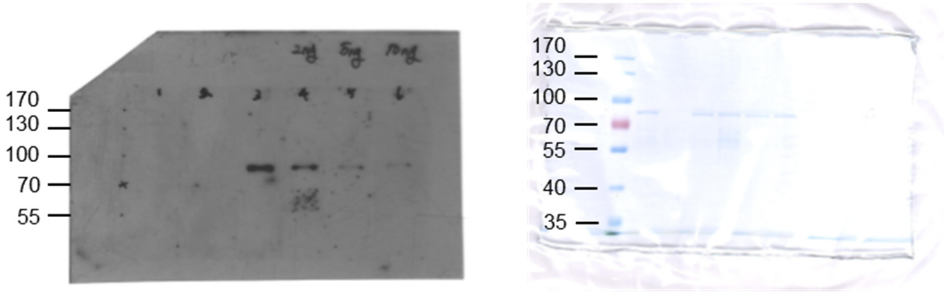
170
130
100
70
55
170
130
100
70
55
40
35

4.C

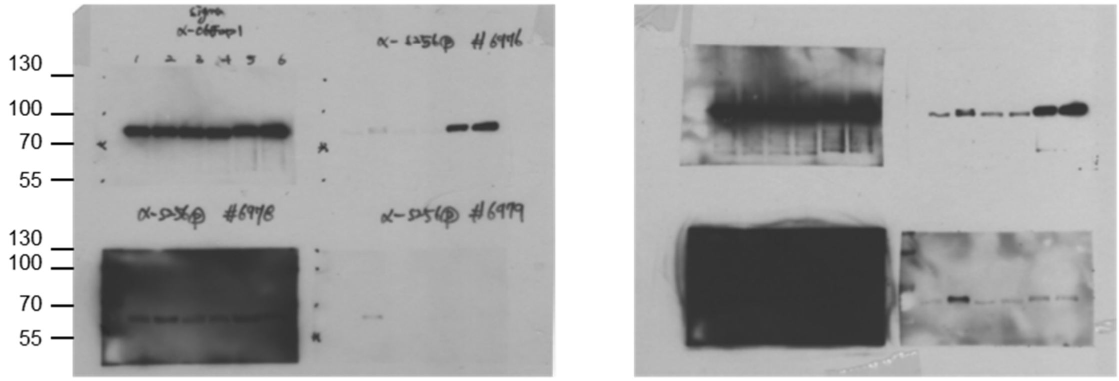
130
100
70
55
130
100
70
55

4.D

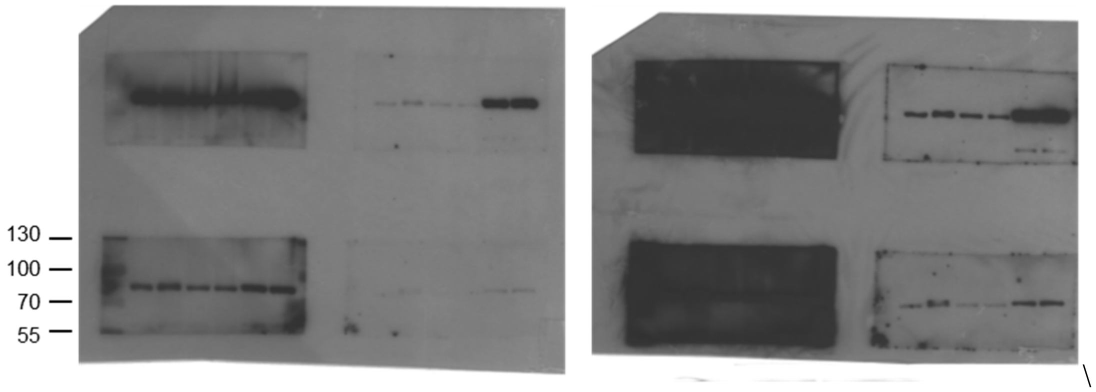

Figure S15. Full western blot of Figure S4.

Table S1. Primer list for knockout, RT-qPCR and ChIP-qPCR.

Name	Sequence
sgRNA oligonucleotides for OGFOD1 knock-out 1-1	CACCGTCAGAAGGAACTGATGAACT
sgRNA oligonucleotides for OGFOD1 knock-out 1-2	AAACAGTTCATCAGTTCCTTCTGAC
sgRNA oligonucleotides for OGFOD1 knock-out 2-1	CACCGCTTCTAAGAAGTCTTGGCTT
sgRNA oligonucleotides for OGFOD1 knock-out 2-2	AAACAAGCCAAGACTTCTTAGAAGC
Oligonucletides for OGFOD1 exon 2_F	CCCAAAATTCATGACCATCC
Oligonucletides for OGFOD1 exon 2 _R	GGGGAATAAATACCTGCTGGA
hOGFOD1 S256A mutagenesis_F	CCCCCCATACCTCGGGCCCCTCACATCCCACA
hOGFOD1 S256A mutagenesis_R	TGTGGGATGTGAGGGGCCCGAGGTATGGGGGG
qRT-PCR primer for TP53_F	TCAACAAGATGTTTTGCCAACTG
qRT-PCR primer for TP53_R	ATGTGCTGTGACTGCTTGTAGATG
qRT-PCR primer for PLAUR_F	TGAAGAAGGGCGTCCAAAG
qRT-PCR primer for PLAUR_R	GTGGTGTTGCAGCATTTCAG
qRT-PCR primer for TLR4_F	GGACCTTTCCAGCAACAAGA
qRT-PCR primer for TLR4_R	CAGGGACAGGTCTAAAGAGAGA
qRT-PCR primer for MMP14_F	CGGCCTTCTGTTCCTGATAAA
qRT-PCR primer for MMP14_R	AGCGCTCCTTGAAGACAAA
qRT-PCR primer for ADAM8_F	GGGCCTGGAGATTTGGAATAG
qRT-PCR primer for ADAM8_R	CGTGATGAGCTGTACGTTGT
qRT-PCR primer for TLR2_F	CCTACTGGGTGGAGAACCTTAT
qRT-PCR primer for TLR2_R	CAGGAATGAAGTCCCGCTTATG
qRT-PCR primer for PLAU_F	CGCATGACTTTGACTGGAATTG
qRT-PCR primer for PLAU_R	TCCAGGGTAAGAAGTGTGAGA
qRT-PCR primer for FZD1_F	AAGACCGAGTGGTGTGTAATG
qRT-PCR primer for FZD1_R	TGGCCATGCTGAAGAAGTAG
qRT-PCR primer for FZD8_F	CATCGGCTACAACTACACCTA
qRT-PCR primer for FZD8_R	CTGCACAGGAAGAACTTGAGA
qRT-PCR primer for BCAR1_F	ACTATGACTACGTCCACCTACA

qRT-PCR primer for BCAR1_R	CTCCTGTTCCAGTCGTTCAAA
qRT-PCR primer for ITGA1_F	GGAAAGAGTATGCACAACGTATTC
qRT-PCR primer for ITGA1_R	CTGTCAGACCGTCACCATTTA
qRT-PCR primer for ITGA2_F	CTGTTCAAGGAGGAGACAACTT
qRT-PCR primer for ITGA2_R	CTGCACCCAGCATCAGAATA
qRT-PCR primer for ITGAV_F	ACTCTTAGCTGGTCTTCGTTTC
qRT-PCR primer for ITGAV_R	TGTGAGATACAACTGGGCTTAC
qRT-PCR primer for ITGAB1_F	AATTAGGCCTCTGGGCTTTAC
qRT-PCR primer for ITGAB1_R	CCTTCATGACACTTGGGACTT
qRT-PCR primer for ITGB2_F	GTGACCAGTAGGATGGTGAAG
qRT-PCR primer for ITGB2_R	GACCCTGGAGGAGAGTTTATTG
qRT-PCR primer for EMP1_F	GCCAATGTCTGGTTGGTTTC
qRT-PCR primer for EMP1_R	CACTGTCTTGAGGGCATCTT
qRT-PCR primer for ARHGAP18_F	GCTCAGTGTGGAGTATCTCAAAG
qRT-PCR primer for ARHGAP18_R	TCAGGTAGGAGGATGACAAGAA
qRT-PCR primer for CTGF_F	GCTGACCTGGAAGAGAACATTA
qRT-PCR primer for CTGF_R	CTCCACAGAATTTAGCTCGGTAT
qRT-PCR primer for GNC11_F	TGCAGAGACAACAAGTGTCTAA
qRT-PCR primer for GNC11_R	TGTCTTCTGGAATTCCCTTTACT
qRT-PCR primer for KISS1_F	AGAAGGACCTGCCGAACTA
qRT-PCR primer for KISS1_R	CTTCTGCCGTGGTTCCC
qRT-PCR primer for DUSP1_F	TTTGAGAACGCAGGAGAGTTTA
qRT-PCR primer for DUSP1_R	TGTACCAAGACACCACAGTTC
ChIP primer for TP53_1_F	CTCTAGCCAAGCTTCCATCC
ChIP primer for TP53_1_R	CTGAACTTGATGAGTCCTCTCTG
ChIP primer for TP53_2_F	CTCCCGAATAGCTGGGATTAC
ChIP primer for TP53_2_R	GGGCGGATTACTTGAGGATAG
ChIP primer for TP53_3_F	TGTGACTGCTTGTAGATGGC
ChIP primer for TP53_3_R	TCTGTCTCCTTCCTCTTCCTAC
ChIP primer for TP53_4_F	CCTGGTTAGTACGGTGAAGTG
ChIP primer for TP53_4_R	AGGGATGTTTGGGAGATGTAAG
ChIP primer for TP53_5_F	CCAGGTATCCTGCCACTTTC
ChIP primer for TP53_5_R	CAGCCTCTCACCAAGGATTAC
ChIP primer for PLAUR_1_F	CAGGTGTGGAGCAGCAG
ChIP primer for PLAUR_1_R	AAACTGCCTCCTTCCTGAG
ChIP primer for PLAUR_2_F	CATCTGAGGTCAGGAGATTGAG
ChIP primer for PLAUR_2_R	TCCTGGGTAGCTGGGATTA
ChIP primer for PLAUR_3_F	GGTAACGGCTTCGGGAATAG
ChIP primer for PLAUR_3_R	GGGTGGGATAAGGAGGAATTG
ChIP primer for PLAUR_4_F	GTGATGGTGAGGCTGAGATG
ChIP primer for PLAUR_4_R	CATTGATGTCTCCTGCTGTACT
ChIP primer for PLAUR_5_F	CGTCCGGATTTAGCCGATTT
ChIP primer for PLAUR_5_R	GGCTGCCTCCACATCTATTT
